# Endocannabinoid levels in plasma and neurotransmitters in the brain: a preliminary report on patients with a psychotic disorder and healthy individuals

**DOI:** 10.1017/S0033291724000291

**Published:** 2024-07

**Authors:** Carmen F. M. van Hooijdonk, Michiel G. J. Balvers, Marieke van der Pluijm, Charlotte L. C. Smith, Lieuwe de Haan, Anouk Schrantee, Maqsood Yaqub, Renger F. Witkamp, Elsmarieke van de Giessen, Therese A. M. J. van Amelsvoort, Jan Booij, Jean-Paul Selten

**Affiliations:** 1 Department of Psychiatry and Neuropsychology, School for Mental Health and Neuroscience (MHeNs), University of Maastricht, Maastricht, The Netherlands; 2 Rivierduinen, Institute for Mental Health Care, Leiden, The Netherlands; 3 Division of Human Nutrition and Health, Wageningen University & Research, Wageningen, The Netherlands; 4 Department of Radiology and Nuclear Medicine, Amsterdam UMC, University of Amsterdam, Amsterdam, The Netherlands; 5 Department of Psychiatry, Amsterdam UMC, University of Amsterdam, Amsterdam, The Netherlands; 6 Department of Radiology and Nuclear Medicine, Amsterdam UMC, Vrije Universiteit Amsterdam, Amsterdam, The Netherlands

**Keywords:** 2-AG, anandamide, dopamine, endocannabinoids, GABA, glutamate, neuroimaging, schizophrenia spectrum disorders

## Abstract

**Background:**

Interactions between the endocannabinoid system (ECS) and neurotransmitter systems might mediate the risk of developing a schizophrenia spectrum disorder (SSD). Consequently, we investigated in patients with SSD and healthy controls (HC) the relations between (1) plasma concentrations of two prototypical endocannabinoids (N-arachidonoylethanolamine [anandamide] and 2-arachidonoylglycerol [2-AG]) and (2) striatal dopamine synthesis capacity (DSC), and glutamate and y-aminobutyric acid (GABA) levels in the anterior cingulate cortex (ACC). As anandamide and 2-AG might reduce the activity of these neurotransmitters, we hypothesized negative correlations between their plasma levels and the abovementioned neurotransmitters in both groups.

**Methods:**

Blood samples were obtained from 18 patients and 16 HC to measure anandamide and 2-AG plasma concentrations. For all subjects, we acquired proton magnetic resonance spectroscopy scans to assess Glx (i.e. glutamate plus glutamine) and GABA + (i.e. GABA plus macromolecules) concentrations in the ACC. Ten patients and 14 HC also underwent [^18^F]F-DOPA positron emission tomography for assessment of striatal DSC. Multiple linear regression analyses were used to investigate the relations between the outcome measures.

**Results:**

A negative association between 2-AG plasma concentration and ACC Glx concentration was found in patients (*p* = 0.008). We found no evidence of other significant relationships between 2-AG or anandamide plasma concentrations and dopaminergic, glutamatergic, or GABAergic measures in either group.

**Conclusions:**

Our preliminary results suggest an association between peripheral 2-AG and ACC Glx levels in patients.

## Introduction

Although the use of cannabis is associated with an increased risk of developing psychotic disorders (Howes et al., 2004; Marconi, Di Forti, Lewis, Murray, & Vassos, 2016), the underlying neurobiological mechanisms are still largely unknown. Two major constituents of Cannabis sativa are delta-9-tetrahydrocannabinol (THC) and cannabidiol (CBD). THC is considered the main psychoactive component of cannabis (i.e. producing the intoxicating ‘high’). Moreover, CBD in concentrations much higher than those found in the Cannabis sativa plant might have antipsychotic properties (McGuire et al., 2018). THC and CBD interact with the endocannabinoid system (ECS) (Leweke et al., 2012; Sherif, Radhakrishnan, D'Souza, & Ranganathan, 2016), an endogenous signalling system that employs endogenous fatty acid-based mediators, referred to as endocannabinoids. Of these, *N*-arachidonoylethanolamine (anandamide) and 2-arachidonoylglycerol (2-AG) are the best known. Both prototypical endocannabinoids are released on-demand and target two G protein-coupled cannabinoid receptors: CB_1_ and CB_2_ (Fakhoury, 2017). The CB_1_ receptor is abundantly expressed in neuronal cells, but also in other tissues, where it is located on plasma and mitochondrial membranes (reviewed in Howlett and Abood (2017)). The CB_2_ receptor is expressed in non-neuronal and neuronal cells, including immune cells (Howlett & Abood, 2017) and microglia (Mecha, Carrillo-Salinas, Feliú, Mestre, & Guaza, 2016; Stella, 2010). Anandamide and 2-AG are involved in various signalling mechanisms, including their role as retrograde messengers released from postsynaptic neurons (Fakhoury, 2017).

Accumulating evidence suggests that the ECS plays a role in the pathophysiology of schizophrenia spectrum disorders (SSD) (Bossong & Niesink, 2010; Bossong, Jansma, Bhattacharyya, & Ramsey, 2014), which makes the ECS a potential target for novel treatments. Minichino et al. (2019) demonstrated, in a whole-group analysis, increased anandamide concentrations in the blood and cerebrospinal fluid (CSF) of patients with psychotic disorders compared to healthy controls (HC). A subsequent analysis revealed significantly increased anandamide concentrations in the CSF (but not blood) of different clinical subgroups (i.e. prodromal, first-episode, and multi-episode) compared to HC. Increased CSF levels of anandamide were related to less severe positive and negative symptoms. This is in line with previous findings (Giuffrida et al., 2004) and suggests that engagement of the ECS might act protectively. More specifically, elevated anandamide levels might constitute a compensatory mechanism to reduce striatal hyperdopaminergia, which has been implicated in the pathogenesis of positive symptoms (Leweke et al., 2004; Minichino et al., 2019). A consistent correlation between blood and CSF anandamide concentrations has not been demonstrated (Giuffrida et al., 2004; Reuter et al., 2017). However, endocannabinoids cross the blood-brain barrier (Maccarrone et al., 2006) and peripheral anandamide and 2-AG concentrations may therefore still be relevant for or reflect central brain processes.

Endocannabinoids most likely also play a role in controlling glutamate *N*-methyl-D-aspartate (NMDA) receptor functioning (Li, Yan, Wilson, & Swartzwelder, 2010; Liu, Bhat, Bowen, & Cheng, 2009; Sánchez-Blázquez, Rodríguez-Muñoz, Vicente-Sánchez, & Garzón, 2013), possibly by regulating NR1 C1 subunit expression (Rodríguez-Muñoz, Sánchez-Blázquez, Callado, Meana, & Garzón-Niño, 2017). As hypo-functioning of NMDA receptors on cortical fast-spiking y-aminobutyric acid (GABA)-ergic interneurons and the subsequent excessive release of glutamate (Egerton et al., 2020; Moghaddam & Javitt, 2012) have been implicated in the pathophysiology of psychotic disorders, these findings suggest that the interaction between the ECS and certain neurotransmitter systems might mediate the risk of developing a psychotic disorder.

Consequently, in this preliminary study, we investigated the associations between striatal dopamine synthesis capacity (DSC), as measured by [^18^F]F-DOPA positron emission tomography (PET), and plasma concentrations of anandamide and 2-AG in patients with SSD and HC. We also examined the associations between Glx (i.e. glutamate plus glutamine) and GABA + concentrations (i.e. GABA plus macromolecules) in the anterior cingulate cortex (ACC), as measured by proton magnetic resonance spectroscopy (^1^H-MRS), and anandamide and 2-AG plasma concentrations. We hypothesized that striatal DSC and ACC Glx and GABA + concentrations are negatively correlated with plasma endocannabinoid levels in both groups, as anandamide is thought to provide retrograde inhibition of the dopaminergic system (Minichino et al., 2019) and binding of anandamide and 2-AG to the pre-synaptically located CB_1_ receptor inhibits the release of glutamate and GABA from presynaptic neurons (Schlicker & Kathmann, 2001; Wilson & Nicoll, 2002). Second, as alterations in endocannabinoid functioning have been reported in schizophrenia, we compared the plasma concentrations of anandamide and 2-AG between patients and HC. Although meta-analytic findings indicate unaltered plasma endocannabinoid levels in first-episode psychosis patients (Minichino et al., 2019), we expected to find lower levels in early psychosis patients compared to HC, as this would (theoretically) result in reduced inhibition of the pertinent neurotransmitters in the brain and the subsequent development of psychotic symptoms.

## Methods

### Participants and procedures

We recruited 18 patients who had experienced a first psychotic episode in the past five years and 16 HC, aged 18–50 years. All patients had been diagnosed with SSD by a certified psychiatrist and received antipsychotic treatment. Their cannabis use was assessed using the Composite International Diagnostic Interview (Ter Smitten, Smeets, & Van den Brink, 1998). To obtain a representative cohort, patients and HC were allowed to have ever used cannabis. However, those who met the criteria for a cannabis use disorder were excluded. Details of recruitment and inclusion/exclusion criteria are explained in online Supplementary Methods S1. The authors assert that all procedures contributing to this work comply with the ethical standards of the relevant national and institutional committees on human experimentation and with the Helsinki Declaration of 1975, as revised in 2008. The study was approved by the Medical Ethical Committees of the Amsterdam Medical Centre (NL63410.018.17) and the Leiden University Medical Center (NL72218.058.20). All participants signed written informed consent. PET data of all subjects are previously reported (van Hooijdonk et al., 2023). ^1^H-MRS data of 12 patients and 16 HC are reported in other yet unpublished manuscripts.

The study was performed on 2 or 3 separate days, depending on the availability of the research facilities, and consisted of screening for in- and exclusion criteria, a ^1^H-MRS and [^18^F]F-DOPA scan (the latter was optional for patients), and blood sampling to assess endocannabinoid plasma concentrations. Blood samples were collected on the same day as the PET scan or, if the patient did not undergo PET scanning, on the same day as the ^1^H-MRS scan. We instructed participants to refrain from cannabis 24 h before the scan(s).

### Blood collection and analysis

The procedures for blood collection and analyses are explained in online Supplementary Methods S2. Briefly, venous blood samples were collected into ethylenediaminetetraacetic acid (EDTA) tubes. Given the fluctuations of plasma anandamide and 2-AG levels throughout the day (Hanlon, 2020; Hanlon et al., 2016), we strived to collect all blood samples in the late afternoon. Samples were centrifuged at room temperature. After centrifugation, 1.10 ml of plasma was stored in a −80 °C freezer in the presence of 1.10 μl of a 100 mm phenylmethanesulfonyl fluoride (PMSF) solution. PMSF is a serine protease inhibitor and prevents anandamide breakdown by fatty acid amide hydrolase (FAAH), which could potentially be released by blood cells during the venipuncture. Plasma concentrations (ng/ml) of anandamide and 2-AG were determined using previously published methods that are based on liquid chromatography coupled to tandem mass spectrometry (LC–MS/MS) (Balvers, Verhoeckx, & Witkamp, 2009; Balvers, Wortelboer, Witkamp, & Verhoeckx, 2013). As 2-AG is prone to isomerization to 1-arachidonoylglycerol (1-AG) and as this is unavoidable in plasma, we reported the total concentration of plasma 1-AG plus 2-AG (hereafter labelled as 2-AG).

### ^1^H-MRS acquisition and pre-processing

The ^1^H-MRS procedures and pre-processing steps are explained in online Supplementary Methods S3. In short, magnetic resonance imaging (MRI) images were obtained on a 3T scanner (Phillips, Ingenia Elition X, Best, The Netherlands) with a 32-channel head coil at the Amsterdam UMC, the Netherlands. A structural whole-brain T1-weighted MRI scan was acquired for ^1^H-MRS voxel placement and brain tissue segmentation. To estimate the concentration of Glx, ^1^H-MRS spectra were acquired using a Point Resolved Spectroscopy (PRESS) sequence. Glx was selected as a proxy for glutamatergic functioning, as it is difficult to differentiate glutamate and glutamine signals at 4T or lower magnetic field strengths (Liemburg et al., 2016). Additional ^1^H-MRS spectra were acquired using a Mescher–Garwood Point Resolved Spectroscopy (MEGA-PRESS) sequence, which is optimized to estimate the concentration of GABA (Mullins et al., 2014). For logistic reasons, two MEGA-PRESS sequences with similar scan parameters were used for the patient group (online Supplementary Table S1). GABA concentrations reflect GABA plus macromolecules (GABA + ) concentrations, as macromolecules resonate at the same frequency as GABA (3.0 ppm) (Rothman, Petroff, Behar, & Mattson, 1993). Both ^1^H-MRS voxels were placed in the ACC parallel to the corpus callosum on the sagittal midline (online Supplementary Fig. S2). PRESS spectra were analyzed by use of LCModel version 6.3-1P (Provencher, 1993, 2014) using the basis set of 16 metabolites. To control the flexibility of the spline baseline, the DKNTMN parameter was set to 0.50 (Bhogal et al., 2017). Gannet version 3.1 was used to analyze the MEGA-PRESS spectra (Edden, Puts, Harris, Barker, & Evans, 2014). All metabolite estimates were water referenced. Spectral quality was assessed and metabolite concentrations were corrected for tissue content as described in online Supplementary Methods S3.

### PET/CT acquisition and pre-processing

The PET/computed tomography (CT) procedures and pre-processing steps are explained in online Supplementary Methods S4. One hour before the PET acquisition, participants consumed 150 mg carbidopa and 400 mg entacapone (Hoffman et al., 1992; Sawle et al., 1994). In addition, a low-dose CT scan of the brain was obtained for attenuation correction purposes. A 90-minute dynamic PET scan was subsequently made on a Siemens PET/CT system (Biograph mCT FlowTrue-V-128) immediately after the administration of approximately 185 MBq [^18^F]F-DOPA. We used Patlak graphical analysis (Patlak & Blasberg, 1985) to calculate the influx constant *k*i^cer^ (min^−1^; henceforth described as *k*i^cer^) as a measure of DSC with the grey matter of the cerebellum as reference region. The volumes of interest (i.e. striatum and cerebellum) were identified from the co-registered T1-weighted MRI scan using the Hammers' maximum probability atlas (Hammers et al., 2003). Afterward, linear fitting was conducted on the PET images acquired between 25 and 90 min to obtain a whole-brain parametric image, from which we extracted the *k*i^cer^ of the grey matter (GM) of the whole striatum.

### Statistical analysis

All statistical analyses were performed in IBM SPSS Statistics (version 22). Outliers per group were defined as observations more than 1.50 × interquartile range below the 1st quartile or more than 1.50 × interquartile range above the 3rd quartile. To examine the association between imaging data and plasma concentrations of endocannabinoids across all subjects, we conducted multiple linear regression analyses with one of the imaging outcomes (i.e. *k*i^cer^ of the GM striatum, Glx, or GABA + concentration in the ACC) as the dependent variable. The independent variables were the plasma concentration of anandamide or 2-AG, group (i.e. patient *v.* HC), and the interaction between the group and the endocannabinoid plasma concentration. In the case of outliers, we repeated the analyses without these outliers. Finally, if associations between imaging and blood outcomes were evident, we repeated the multiple linear regression analysis and added one more covariate. Due to the limited sample size, we assessed the effects of age, sex, and cannabis lifetime use separately. This is important because age and sex have an impact on striatal DSC (Kumakura et al., 2005; Nordio et al., 2022) and ACC glutamate concentration (Hädel, Wirth, Rapp, Gallinat, & Schubert, 2013). Moreover, the main constituents of cannabis, THC and CBD, affect the ECS (Leweke et al., 2012). A *p* value of less than 0.05 was considered statistically significant. No correction for multiple testing was applied, as all analyses were preliminary.

Differences between patients and HC with regard to sociodemographic characteristics (e.g. age and sex) and imaging and blood outcomes were examined utilizing a parametric independent *t* test, Mann–Whitney *U* test or Fisher's exact test, depending on the variable type and its distribution.

## Results

### Sample characteristics

Eighteen patients with SSD and 16 HC completed the study. Plasma concentrations of endocannabinoids were determined for all subjects. We were unable to use the [^18^F]F-DOPA data of 3 patients and 2 HC, due to substantial head movements (> 7.50 mm). Five patients did not want to take part in the [^18^F]F-DOPA PET part of the study. Thus, the final PET sample consisted of 10 patients and 14 HC. With regard to the ^1^H-MRS analysis, one patient was excluded because of benzodiazepine use. One additional patient was excluded from the Glx analysis due to a low signal-to-noise ratio (SNR). The final ^1^H-MRS samples consisted of 16 HC, as well as 16 and 17 patients, respectively, for the analyses related to PRESS and MEGA-PRESS. All subjects, except two patients, had a negative drug screen for cannabis use.

There were no significant between-group differences in age, race, educational level, current tobacco use (i.e. at least daily for one month in the past year), or lifetime cannabis use (i.e. ⩽ 5 *v.* > 5 times) in the PET, PRESS, or MEGA-PRESS samples. However, there were numerical differences between groups with regard to educational level, current tobacco use, and lifetime cannabis use (i.e. ⩽ 5 *v.* > 5 times; Table 1). Additionally, patients had significantly higher Beck Depression Inventory scores than HC (PET: *U* = 5.00, *p* < 0.001; PRESS: *U* = 22.00, *p* < 0.001; MEGA-PRES: *U* = 23.00, *p* < 0.001). The patient groups consisted of more male subjects compared to the HC group (PET: *p* = 0.34; PRESS: *p* = 0.02; MEGA-PRESS: *p* = 0.04). Lastly, we found no significant differences in injected [^18^F]F-DOPA dose between patients and HC who underwent PET imaging.

**Table 1.** Sample characteristics

	PET sample			MRI samples				
				PRESS	MEGA-PRESS	PRESS & MEGA-PRESS		
	Patients with SSD (*n* = 10)	Healthy controls (*n* = 14)	*p* Value	Patients with SSD (*n* = 16)	Patients with SSD (*n* = 17)	Healthy controls (*n* = 16)	*p* Value^a^	*p* Value^b^
Demographics								
Age in years, mean (s.d.)^c^	21.30 (2.75)	22.71 (3.95)	0.47^d^	22.94 (4.45)	22.82 (4.33)	23.19 (4.56)	0.92^d^	0.93^d^
No. (%) male sex	9 (90%)	9 (64.29%)	0.34^e^	16 (100%)	16 (94.12%)	10 (62.50%)	**0.02**^e^	**0.04**^e^
No. (%) white race	9 (90%)	13 (92.86%)	1.00^e^	14 (87.50%)	14 (82.35%)	15 (93.75%)	1.00^e^	0.60^e^
No. (%) highest educational degree			0.66^e^				0.39^e^	0.47^e^
Pre-vocational secondary education	0 (0%)	0 (0%)		1 (6.25%)	1 (5.88%)	0 (0%)		
Secondary vocational education	3 (30%)	6 (42.86%)		4 (25%)	5 (29.41%)	7 (43.75%)		
Senior general secondary education / Pre-university education	5 (50%)	2 (14.29%)		6 (37.50%)	6 (35.29%)	2 (12.50%)		
Higher professional education / University education (Bachelor's degree)	2 (20%)	5 (35.71%)		4 (25%)	4 (23.53%)	6 (37.50%)		
University education (Master's degree)	0 (0%)	1 (7.14%)		1 (6.25%)	1 (5.88%)	1 (6.25%)		
No. (%) DSM-V diagnosis		NA	NA			NA	NA	NA
Schizophrenia	5 (50%)			6 (37.50%)	7 (41.18%)			
Schizoaffective disorder	1 (10%)			1 (6.25%)	1 (5.88%)			
Schizophreniform disorder	2 (20%)			3 (18.75%)	3 (17.65%)			
Unspecified schizophrenia spectrum and other psychotic disorder	1 (10%)			3 (18.75%)	3 (17.65%)			
Other specified schizophrenia spectrum and other psychotic disorder	1 (10%)			3 (18.75%)	3 (17.65%)			
PANSS at study enrollment, mean (s.d.)								
Positive score	13.20 (5.81)	NA	NA	12.44 (4.59)	12.88 (4.81)	NA	NA	NA
Negative score	15.20 (7.10)	NA	NA	13.88 (6.22)	13.94 (6.03)	NA	NA	NA
General score	28.00 (8.76)	NA	NA	26.31 (7.27)	26.76 (7.28)	NA	NA	NA
Total score	56.40 (19.53)	NA	NA	52.63 (16.14)	53.59 (16.12)	NA	NA	NA
BDI, mean (s.d.)	13.70 (8.76)	1.50 (1.99)	**<0.001**^d^	10.94 (7.56)	11.29 (7.46)	2.56 (4.76)	**<0.001**^d^	**<0.001**^d^
No. (%) current tobacco users^f^	3 (30%)	3 (21.43%)	0.67^e^	8 (50%)	8 (47.06%)	4 (25%)	0.27^e^	0.28^e^
No. (%) cannabis users^g^	5 (50%)	3 (21.43%)	0.20^e^	10 (62.50%)	10 (58.82%)	4 (25%)	0.07^e^	0.08^e^
Injected [^18^F]F-DOPA dose in MBq, mean (s.d.)	179.96 (13.75)	182.31 (15.46)	0.55^d^	NA	NA	NA	NA	NA
Imaging measures								
Striatal DSC in min-1, mean (s.d.)	0.0159 (0.0026)	0.0170 (0.0011)	0.24^h^^,^^i^	NA	NA	NA	NA	NA
Glx in the ACC, mean (s.d.)	NA	NA	NA	19.06 (1.36)	NA	18.99 (1.14)	0.87^h^	NA
GABA + in the ACC, mean (s.d.)	NA	NA	NA	NA	2.86 (0.48)	3.43 (0.45)	NA	**<0.01**^h^^,^^j^
**Blood measures**	**Patients with SSD (*n* = 18)**	**Healthy controls (*n* = 16)**	***p* Value**					
Anandamide plasma concentration in ng/ml, mean (s.d.)	0.31 (0.11)	0.43 (0.11)	**<0.01**^d^	NA	NA	NA	NA	NA
2-AG plasma concentration in ng/ml, mean (s.d.)	3.53 (2.95)	3.07 (1.98)	0.75^d^^,^^k^	NA	NA	NA	NA	NA

*Abbreviations*: ACC, anterior cingulate cortex; BDI, Beck Depression Inventory; CPZ, chlorpromazine; DSC, dopamine synthesis capacity; DSM-V, Diagnostic and Statistical Manual of Mental Disorders, Fifth Edition; GABA, y-aminobutyric acid; Glx, glutamate plus glutamine; MBq, megabecquerel; MEGA-PRESS, Mescher-Garwood Point Resolved Spectroscopy; NA, not applicable; PANSS, positive and negative symptom scale; PET, positron emission tomography; PRESS, Point Resolved Spectroscopy; s.d., standard deviation; SSD, schizophrenia spectrum disorder; 2-AG, 2-arachidonoylglycerol.

Significant results are bold.

a Group differences for the PRESS sample.

b Group differences for the MEGA-PRESS sample.

c During the PET or MRI scan.

d Group differences were assessed with the Mann–Whitney *U* test.

e Group differences were assessed with Fisher's exact test.

f Current tobacco use was defined as having used tobacco daily for at least one month in the past twelve months.

g Cannabis use was defined as having used cannabis at least six times in a lifetime.

h Group differences were assessed with an independent *t* test.

i One outlier was identified (i.e. one patient). After the exclusion of this outlier, significant group differences were found (online Supplementary Table S4).

j After correction for SNR for GABA+, the difference between groups did not remain significant (*p* = 0.27).

k Four outliers were identified (i.e. two patients and two healthy controls). Excluding these subjects did not change the results (online Supplementary Table S4).

While the plasma concentrations of 2-AG did not differ significantly between the two groups, those of anandamide were significantly lower in patients than in HC (*p* < 0.01; Table 1). This difference remained significant after adjusting for sex and lifetime cannabis use (i.e. ⩽ 5 *v.* > 5 times) (*p* = 0.047; online Supplementary Table S3). Initially, we found lower ACC GABA + concentrations in patients than in HC (*p* < 0.01). However, the two different MEGA-PRESS sequences to assess GABA + concentration in the patient group yielded different mean SNRs for GABA + . After adding SNR as covariate, the GABA + difference between patients and HC was no longer significant (*p* = 0.27). Remarkably, after the exclusion of one outlier, we found a lower striatal DSC (*p* < 0.01) in patients compared to HC (online Supplementary Table S4). Group had no significant impact on Glx concentration in the ACC. The group differences in endocannabinoid plasma levels and Glx and GABA concentrations in the ACC did not change significantly after the exclusion of two patients who had a positive drug screen for cannabis.

### Multiple linear regression analyses: anandamide

A multiple linear regression analysis while correcting for group revealed that anandamide plasma concentration was positively associated with striatal DSC in patients, but not in HC (patients: unstandardized beta = 0.01, *p* = 0.02; HC: unstandardized beta = 0.0002, *p* = 0.96; Table 2; online Supplementary Fig. S3). However, this association did not remain significant after the removal of one outlier. The interaction between group and anandamide plasma concentration was of borderline significance (unstandardized beta = 0.01, *p* = 0.08), although clearly non-significant after the removal of one outlier (unstandardized beta = 0.003, *p* = 0.58; Table 2). In addition, we found no significant associations while correcting for group between anandamide plasma concentration and Glx or GABA + concentration in the ACC in patients or HC (Table 2). There was also no evidence that these associations might differ between groups. These results did not change after excluding two patients with a positive drug screen for cannabis (online Supplementary Table S5) or when adding SNR for GABA + as covariate (online Supplementary Table S6). See online Supplementary Fig. S3 for scatterplots displaying the association between anandamide and imaging measures.

**Table 2.** Multiple linear regression analyses between imaging and blood measures

Independent variables	Unstandardized beta coefficients	*p* value
*Model 1a*: Dependent variable: striatal DSC, outliers *not* excluded^a^, *R*^2^ = 0.31, *F*_(3, 20)_ = 2.96, *p* value = 0.06		
Group^b^	−0.004 [−0.009 to 0.0005]	0.07
Effect of anandamide plasma concentration in HC	0.0002 [−0.008 to 0.009]	0.96
Effect of anandamide plasma concentration in patients	0.01 [0.002–0.02]	**0.02**
Interaction term: group × anandamide plasma concentration	0.01 [−0.002 to 0.02]	0.08
*Model 1b*: Dependent variable: striatal DSC, outliers excluded^a^, *R*^2^ = 0.33, *F*_(3, 19)_ = 3.12, *p* value = 0.051		
Group^b^	−0.003 [−0.007 to 0.002]	0.21
Effect of anandamide plasma concentration in HC	0.0002 [−0.007 to 0.007]	0.95
Effect of anandamide plasma concentration in patients	0.003 [ −0.006 to 0.01]	0.46
Interaction term: group × anandamide plasma concentration	0.003 [−0.008 to 0.01]	0.58
*Model 2a*: Dependent variable: striatal DSC, outliers *not* excluded^a^, *R*^2^ = 0.09, *F*_(3, 20)_ = 0.64, *p* value = 0.60		
Group^b^	−0.001 [−0.005 to 0.003]	0.49
Effect of 2-AG plasma concentration in HC	−0.00009 [−0.001 to 0.001]	0.77
Effect of 2-AG plasma concentration in patients	−0.00002 [−0.001 to 0.001]	0.97
Interaction term: group × 2-AG plasma concentration of 2-AG	000008 [−0.001 to 0.001]	0.89
*Model 2b*: Dependent variable: striatal DSC, outliers excluded^a^, *R*^2^ = 0.42, *F*_(3, 17)_ = 4.13, *p* value = **0.02**		
Group^b^	−0.002 [−0.006 to 0.002]	0.34
Effect of 2-AG plasma concentration in HC	−0.00003 [−0.001 to 0.001]	0.93
Effect of 2-AG plasma concentration in patients	−0.0001 [ −0.001 to 0.001]	0.86
Interaction term: Group × 2-AG plasma concentration of 2-AG	−0.00008 [−0.001 to 0.001]	0.91
*Model 3*: Dependent variable: Glx in the ACC, no outliers, *R*^2^ = 0.10, *F*_(3, 28)_ = 1.07, *p* value = 0.38		
Group^b^	1.34 [−1.91 to 4.58]	0.41
Effect of anandamide plasma concentration in HC	−0.96 [−6.62 to 4.70]	0.09
Effect of anandamide plasma concentration in patients	−5.73 [−4.58 to 1.91]	0.09
Interaction term: Group × Anandamide plasma concentration	−4.77 [−13.53 to 4.00]	0.28
*Model 4a*: Dependent variable: Glx in the ACC, outliers *not* excluded^a^, *R*^2^ = 0.09, *F*_(3, 28)_ = 0.90, *p* value = 0.45		
Group^b^	0.71 [−0.87 to 2.28]	0.37
Effect of 2-AG plasma concentration in HC	−0.003 [−0.33 to 0.33]	0.99
Effect of 2-AG plasma concentration in patients	−0.17 [−0.39 to 0.04]	0.11
Interaction term: Group × 2-AG plasma concentration	−0.17 [−0.56 to 0.23]	0.39
*Model 4b*: Dependent variable: Glx in the ACC, outliers excluded^a^, *R*^2^ = 0.29, *F*_(3, 24)_ = 3.22, *p* value = **0.04**		
Group^b^	3.24 [0.91–5.56]	**0.008**
Effect of 2-AG plasma concentration in HC	0.25 [−0.22 to 0.72]	0.29
Effect of 2-AG plasma concentration in patients	−0.92 [−1.58 to −0.27]	**0.008**
Interaction term: Group × 2-AG plasma concentration	−1.17 [−1.97 to −0.37]	**0.006**
*Model 5*: Dependent variable: GABA + in the ACC, no outliers, *R*^2^ = 0.31, *F*_(3, 29)_ = 4.36, *p* value = **0.01**		
Group^b^	−0.61 [−1.81 to 0.60]	0.31
Effect of anandamide plasma concentration in HC	−0.76 [−2.94 to 1.41]	0.48
Effect of anandamide plasma concentration in patients	−0.94 [−3.18 to 1.31]	0.40
Interaction term: group × anandamide plasma concentration	−0.17 [−3.30 to 2.95]	0.91
*Model 6a*: Dependent variable: GABA + in the ACC, outliers *not* excluded^a^, *R*^2^ = 0.38, *F*_(3, 29)_ = 5.98, *p* value = **0.003**		
Group^b^	−0.87 [−1.43 to −0.31]	**0.003**
Effect of 2-AG plasma concentration in HC	−0.12 [−0.24 to −0.004]	**0.04**
Effect of 2-AG plasma concentration in patients	−0.02 [−0.10 to 0.06]	0.59
Interaction term: Group × 2-AG plasma concentration	0.10 [−0.04 to 0.24]	0.15
*Model 6b*: Dependent variable: GABA + in the ACC, outliers excluded^a^, *R*^2^ = 0.47, *F*_(3, 25)_ = 7.25, *p* value = **0.001**		
Group^b^	−0.06 [−0.98 to 0.86]	0.89
Effect of 2-AG plasma concentration in HC	−0.08 [−0.27 to 0.12]	0.42
Effect of 2-AG plasma concentration in patients	−0.28 [−0.54 to −0.02]	**0.04**
Interaction term: Group × 2-AG plasma concentration	−0.20 [−0.53 to 0.12]	0.21

*Abbreviations*: ACC, anterior cingulate cortex; DSC, dopamine synthesis capacity; GABA + , y-aminobutyric acid plus macromolecules; Glx, glutamate plus glutamine; HC, healthy controls, 2-AG, 2-arachidonoylglycerol.

Significant results are bold.

a For the PET data, one outlier was identified. For the 2-AG data, four outliers were identified.

b Group was coded as null for healthy controls and one for patients. To examine the effects in the patient group, reversed coding for group was used.

### Multiple linear regression analyses: 2-AG

We found no significant association between 2-AG plasma concentration and striatal DSC in patients or HC, while correcting for group (Table 2). This did not change after the removal of three outliers (2 of 10 patients and 1 of 14 HC). The GABA + concentration in the ACC was negatively associated with 2-AG plasma concentration in HC, but not in patients, while correcting for group (patients: unstandardized beta = −0.02, *p* = 0.59; HC: unstandardized beta = −0.12, *p* = 0.04). After removal of four outliers, we found a significant negative association in patients, but not in HC (patients: unstandardized beta = −0.28, *p* = 0.04; HC: unstandardized beta = −0.08, *p* = 0.42). The interaction term did not reach significance, indicating that the associations between GABA + concentration in the ACC and 2-AG plasma concentration might not differ between groups. We found no significant associations between 2-AG and GABA + in any of the groups after correcting for SNR for GABA + (online Supplementary Table S6).

**Figure 1.**
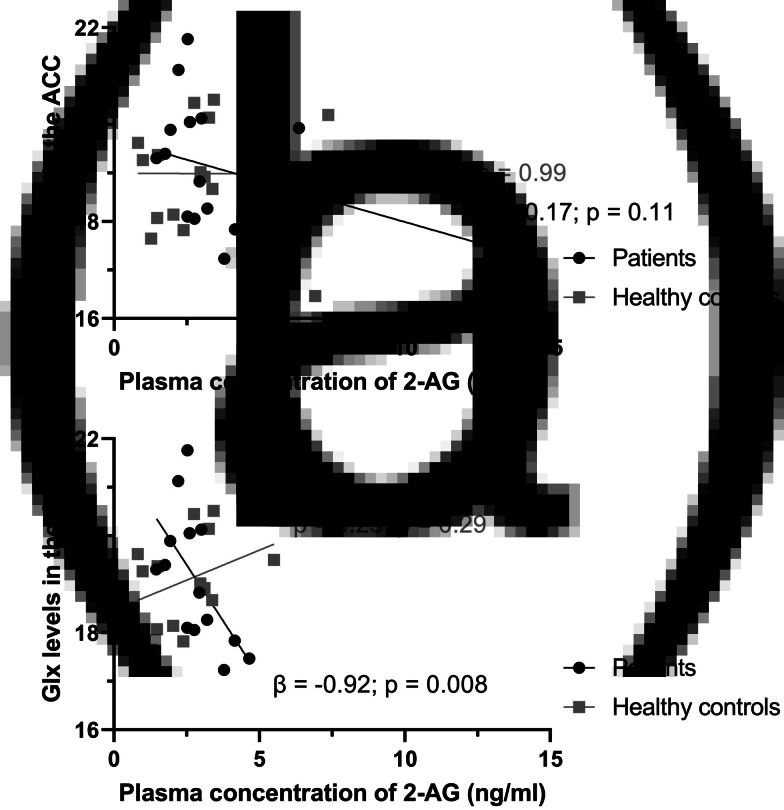
2-AG and Glx in patients with SSD and controls. Scatterplot displaying the correlation between 2-AG plasma concentration and Glx levels in the ACC in patients with SSD (black circles) and healthy controls (grey squares). (a) Outliers are not excluded. (b) Four outliers (2 patients and 2 healthy controls) are excluded.#*Abbreviations*: ACC, anterior cingulate cortex; Glx, glutamate plus glutamine; SSD, schizophrenia spectrum disorders; 2-AG, 2-arachidonoylglycerol; *β*, unstandardized beta.

2-AG plasma concentration was not associated with Glx concentration in the ACC of patients or HC, while correcting for group (Table 2). However, after the removal of four outliers and while correcting for group, 2-AG plasma concentration was negatively associated with Glx concentration in the ACC of patients (unstandardized beta = −0.92, *p* = 0.02; Fig. 1). This was not found in HC (unstandardized beta = 0.25, *p* = 0.29). The interaction between group and 2-AG plasma concentration was significantly associated with Glx concentration in the ACC (unstandardized beta = −1.17, *p* = 0.006), which suggests that the associations between Glx concentration in the ACC and 2-AG plasma concentration differ between the two groups. These results did not change after excluding two patients with a positive drug screen for cannabis (online Supplementary Table S5). See online Supplementary Fig. S4 for scatterplots displaying the association between 2-AG and other imaging measures.

To examine whether the association between Glx concentration in the ACC and 2-AG plasma concentration might be explained by the effects of other variables, we performed additional multiple linear regression analyses, after the removal of outliers, with age, sex, and cannabis lifetime use separately added to the model. In all models, the negative association between Glx concentration in the ACC and 2-AG plasma concentration in patients remained evident, while controlling for group and age, sex, or cannabis lifetime use (online Supplementary Table S7). In addition, the interaction term between group and 2-AG plasma concentration remained significant in all models, suggesting that the relationships between Glx concentration in the ACC and 2-AG plasma concentration are significantly different in patients and HC, also after controlling for additional confounders. These results did not change after excluding two patients with a positive drug screen for cannabis (online Supplementary Table S8).

## Discussion

We used [^18^F]F-DOPA PET, ^1^H-MRS, and LC–MS/MS to examine the interrelationships between plasma endocannabinoid levels and dopaminergic, glutamatergic, and GABAergic measures in patients with SSD and HC. The results of our analyses suggested a significant negative association between Glx concentrations in the ACC and 2-AG plasma concentrations in patients (after the exclusion of probable outliers), but no such association in HC. 2-AG plasma concentrations were not consistently related to GABA + concentrations in the ACC or striatal DSC, neither in patients nor in HC. We found no evidence for relationships between anandamide plasma concentrations and dopaminergic, glutamatergic, or GABAergic measures in either group. Furthermore, contrary to our expectations, we found lower anandamide plasma concentrations and lower striatal DSC in patients compared to controls.

The negative correlation between Glx concentrations in the ACC and 2-AG plasma concentrations in patients was in line with our hypothesis, as binding of 2-AG to the CB_1_ receptor inhibits the release of glutamate and GABA from presynaptic neurons (Wilson & Nicoll, 2002). As excessive glutamate concentrations are neurotoxic, it has been hypothesized that disinhibition of glutamatergic projections to hippocampal and cortical areas cause cell death in some patients with schizophrenia (Deutsch, Rosse, Schwartz, & Mastropaolo, 2001). If this is true, endocannabinoids might serve as endogenous mediators of neuroprotection. Astrocytes also play a role herein, as these cells can take up glutamate and thereby regulate the extracellular concentration of glutamate. In Sprague–Dawley rats, the expression of astrocytic glutamine synthetase, an astrocyte-specific enzyme that converts glutamate to glutamine (Bak, Schousboe, & Waagepetersen, 2006), is modulated by 2-AG following lipopolysaccharide-induced inflammation (Wang et al., 2018; Wang, Wang, & Zhang, 2021). In addition, the activation of metabotropic and ionotropic glutamatergic receptors triggers the production of endocannabinoids, such as 2-AG, in several brain regions (Chevaleyre & Castillo, 2003; Galante & Diana, 2004; Maejima, Hashimoto, Yoshida, Aiba, & Kano, 2001; Ohno-Shosaku, Shosaku, Tsubokawa, & Kano, 2002). These findings suggest that 2-AG can induce compensatory mechanisms to reduce a possibly increased release of glutamate in patients. We found no evidence of an association between ACC Glx levels and 2-AG plasma concentrations in HC. Possibly, a compensatory mechanism to reduce glutamatergic neurotransmission is only triggered when needed, as endocannabinoids are released upon demand.

We found no associations between anandamide plasma concentrations and Glx in the ACC in either group. Watts et al. (2022) measured the activity of the enzyme FAAH, which breaks down anandamide, by use of [^11^C]CURB PET. They demonstrated a positive association between FAAH activity (in eight different brain regions, including the ACC) and Glx concentration in the hippocampus of patients with psychotic disorders (*n* = 18) and HC (*n* = 19). Although additional research is needed, this might indicate that the association between peripheral anandamide and Glx is dependent on the brain region where Glx is measured. Moreover, as 2-AG has been found in the brain at levels 170 times greater than anandamide (Stella, Schweitzer, & Piomelli, 1997), it might play a more important role than anandamide in the self-regulatory mechanisms of glutamatergic neurons in the frontal cortex. This hypothesis needs to be validated by future research.

Glutamate, glutamine, and GABA are closely related to each other by a sequence of events, known as the glutamate/GABA-glutamine cycle (Bak et al., 2006). Previous research showed that both 2-AG and anandamide act as positive allosteric modulators of GABA_A_ receptors (Baur et al., 2013; Sigel et al., 2011). Bakas et al. (2017) demonstrated that, in the case of 2-AG, this results in an increased affinity of GABA for the GABA_A_ receptor at low GABA concentrations. We report a negative correlation between GABA + concentration in the ACC and 2-AG plasma concentration in HC, but not in patients. However, after the removal of outliers, we found a negative correlation between these two measures in patients, but not in HC. The discrepancy between these analyses indicates that a few observations have a large influence on the outcome. Moreover, the negative correlation in patients did not remain significant after removing two patients with a positive drug screen for cannabis (online Supplementary Table S5) and we found no significant correlations after correction for the SNR for GABA + (online Supplementary Table S6). Therefore, additional research in larger samples is needed to elucidate the relationship between peripheral 2-AG (and anandamide) levels and GABA + concentrations in the ACC.

Although endocannabinoids control dopaminergic neurotransmission in the striatum and midbrain (Covey, Mateo, Sulzer, Cheer, & Lovinger, 2017), we found no association between 2-AG plasma concentrations and striatal DSC (i.e. an indicator of striatal hyperdopaminergia). Contrary to expectation, we even found a positive association between anandamide plasma concentration and striatal DSC in patients, but this relationship did not remain significant after the removal of one outlier. However, given our relatively small sample, it is too early to rule out significant associations between these measures in patients or HC.

We found lower anandamide plasma concentrations in patients than in HC. This is not in line with the meta-analysis of Minichino et al. (2019), which reported an elevation of anandamide across clinical subgroups of patients with schizophrenia compared to HC and, in an additional analysis, no significant alterations in first-episode patients. Minichino et al. (2019) also proposed that antipsychotic medication downregulates anandamide concentrations in blood. As the patients in our sample all used antipsychotic medication, this might explain our finding. Alternatively, lower levels of anandamide might be truly evident in early psychosis (antipsychotic-naïve) patients compared to HC, as this would (theoretically) result in reduced inhibition of several important neurotransmitters in the brain and the subsequent development of psychotic symptoms. It is also possible, however, that anandamide plasma levels fluctuate more according to the degree of a specific psychotic episode (acute deterioration *v.* remission) than the nature of the disorder (e.g. early psychosis, remitting-relapsing stage, continuous residual symptoms). Finally, an increase in anandamide might reflect a compensatory mechanism in some patients. Additional research is needed to investigate these hypotheses. Our finding of no significant difference between patients and HC with regard to 2-AG plasma concentration is in line with a report by Potvin et al. (2008).

This study has several strengths. It combines multiple neuroimaging techniques and it is the first to examine, *in vivo*, the association between several neurotransmitter systems and endocannabinoid plasma concentrations in patients with SSD and HC. However, some limitations should be mentioned. First, the sample size is small, especially for the subgroups that underwent PET scanning. The small sample size probably also explains the discrepancies between analyses with and without outliers. Second, the association between Glx concentrations in the ACC and 2-AG plasma concentrations in patients emerged after the exclusion of four outliers, while there was no absolute certainty that the pertinent patients were true outliers. Excluding outliers would preferably be supported by technical, biological, and/or other reasons. The *k*i^cer^ distribution of 892 scans reported by Nordio et al. (2022), for instance, supported the exclusion of the DSC outlier. Likewise, one 2-AG outlier, with a negative drug screen, had used cannabis for 26 weeks in the year before study participation. Other outliers may be explained by unknown pre-analytic or biological factors. Third, endocannabinoid plasma concentrations are influenced by, among others, antipsychotic medication (Minichino et al., 2019), cognitive performance (Reuter et al., 2017), cannabis use (Chester et al., 2022), sleep and circadian rhythm (Hanlon, 2020; Hanlon et al., 2016), nutrition and body mass index (Jager & Witkamp, 2014; Joosten, Balvers, Verhoeckx, Hendriks, & Witkamp, 2010), menstrual cycle (Dlugos, Childs, Stuhr, Hillard, & De Wit, 2012), inflammation (Hillard, 2018), physical exercise (Hillard, 2018), and blood collection and storage procedures. It is unlikely that cannabis use influenced the observed results, as we excluded subjects with a cannabis use disorder and the sensitivity analyses without two patients with a positive drug screen for cannabis resulted in similar findings. Due to the small sample size, we could only adjust our main findings for age, sex, and cannabis lifetime use. It is, therefore, important that the above-described factors are taken into account while setting up future, larger, studies in this research area. Fourth, this study was cross-sectional. Future studies should examine, for example, whether alterations in the ECS precede or succeed alterations in neurotransmitter systems in psychosis. Fifth, the sources of the peripheral endocannabinoids that we measured are unknown and CSF anandamide concentrations do not correlate consistently with anandamide concentrations in blood (Giuffrida et al., 2004; Reuter et al., 2017). Therefore, peripheral concentrations of endocannabinoids might not reflect those in a specific brain region but an average of endocannabinoids in the whole brain. Sixth, as it is difficult to differentiate glutamate and glutamine signals at 4T or lower magnetic field strengths (Liemburg et al., 2016), we used the composite measure Glx. Future MRI studies at higher field strengths should examine whether endocannabinoid plasma concentrations are differently related to glutamine and glutamate. Finally, although the scan parameters of the two utilized MEGA-PRESS sequences were similar, differences in SNR were noticeable within the patient group.

## Conclusions

These preliminary results suggest an association between peripheral 2-AG and ACC Glx levels in patients with SSD. This might reflect a mechanism to compensate for a possible increase of neurotoxic glutamate in patients and points to the ECS as a potential target for novel treatments. More research in larger cohorts is needed to replicate our findings.

## Supporting information

van Hooijdonk et al. supplementary material van Hooijdonk et al. supplementary material

## Supplementary material

The supplementary material for this article can be found at https://doi.org/10.1017/S0033291724000291
