# Supplementary material for: Endocannabinoid levels in plasma and neurotransmitters in the brain: a preliminary report on patients with a psychotic disorder and healthy individuals
Source: Psychol Med. 2024 Feb 23;54(9):2189–99. doi: 10.1017/S0033291724000291 (PMC11413354; doi:10.1017/S0033291724000291)
Supplement: van Hooijdonk et al. supplementary material [file S0033291724000291sup001.docx]

**Supplementary material for:**

***Endocannabinoid levels in plasma and neurotransmitters in the brain: a preliminary report on patients with a psychotic disorder and healthy individuals***

*van Hooijdonk CFM, Balvers MGJ, van der Pluijm M, Smith C, de Haan L, Schrantee A, Yaqub M, Witkamp RF, van de Giessen E, van Amelsvoort TAMJ, Booij J, Selten JP.*

**Table of contents:**

*Supplementary methods*

Methods S1: Recruitment and study criteria.

Methods S2: Blood analysis.

Methods S3: ^1^H-MRS acquisition and pre-processing.

Methods S4: PET acquisition and pre-processing.

*Supplementary tables*

Table S1: Description of MRS data acquisition, analysis, and quality assessment.

Table S2: Spectral quality and tissue fraction measures.

Table S3: Anandamide group differences.

Table S4: Group differences after the exclusion of outliers.

Table S5: Multivariate linear regression analyses between imaging and blood measures without two patients that tested positive on cannabis.

Table S6: Additional multivariate linear regression analyses with signal to noise ratio for GABA+ as covariate.

Table S7: Additional multivariate linear regression analyses to examine the effect of covariates.

Table S8: Additional multivariate linear regression analyses to examine the effect of covariates without two patients that tested positive on cannabis.

*Supplementary figures*

Figure S1: Example ^1^H-MRS spectra of one healthy control.

Figure S2: ^1^H-MRS voxel placement.

Figure S3: Anandamide and imaging measures in patients with SSD and controls.

Figure S4: 2-AG and imaging measures in patients with SSD and controls.

*Supplementary methods*

**Methods S1**. Recruitment and study criteria.

Recruitment:

Patients with a schizophrenia spectrum disorder were recruited via three Dutch mental health institutes (i.e., Rivierduinen Institute for Mental Health Care in Leiden, GGZ inGeest Specialized Mental Health Care in Amsterdam, and the specialized Early Psychosis Clinic of the Amsterdam UMC in Amsterdam). Healthy controls (HC) were recruited via social media.

Inclusion criteria for patients and HC:

- 18-50 years of age.

Inclusion criteria for patients only:

- Diagnostic and Statistical Manual of Mental Disorders, Fifth Edition (DSM-5) diagnosis of a schizophrenia spectrum disorder (i.e., schizophrenia, schizoaffective disorder, schizophreniform disorder, brief psychotic disorder, other specified schizophrenia spectrum and other psychotic disorder, or unspecified schizophrenia spectrum and other psychotic disorder). We confirmed all diagnoses by use of the semi-structured Comprehensive Assessment of Symptoms and History (CASH) interview (Andreasen, 1987).

Exclusion criteria for patients only:

- Onset of first psychotic episode longer than five years ago.
- Previous antipsychotic use longer than one year.
- Use of benzodiazepines on the day of the magnetic resonance imaging (MRI) scan. These patients will only be excluded from the proton magnetic resonance spectroscopy (^1^H-MRS) analysis.

Exclusion criteria for HC only:

- Current psychiatric disorder or history of any psychiatric disorder as assessed with the Mini International Neuropsychiatric Interview (Sheehan *et al.*, 1998).
- First-degree relative with a schizophrenia spectrum disorder.

Additional exclusion criteria for both groups:

- Current disorder in the use of substances other than alcohol or nicotine (or a history of such a disorder), as assessed with the Composite International Diagnostic Interview (CIDI) (Ter Smitten, Smeets, & Van den Brink, 1998).
- Use of substances other than alcohol, cannabis, or nicotine (i.e., psychotropic medication or illicit drugs that influence the dopaminergic system) during the period of three months (in case of patients) or of one month (in case of HC) before study participation. Patients were allowed to use hypnotics, antidepressants, and benzodiazepines in amounts within the therapeutic range.
- Positive urine drug screen on the day of the MRI or positron emission tomography (PET) scan. Participants were tested on the use of XTC, amphetamine, cannabis, opiates, and cocaine. Only a positive drug screen for cannabis use was permitted. This was done as many patients use cannabis (and nicotine), and, therefore, excluding them would lead to a non-representative cohort.
- Positive urine pregnancy screen on the day of the MRI or PET scan (females only).
- Evidence of brain damage or neurological disorders (e.g., epilepsy).
- Contra-indications for MRI or PET (e.g., a pacemaker or ferromagnetic implants).
- Participation in a scientific examination where radiation was used in the year before study participation.
- Inability to give informed consent.

**Methods S2**. Blood analysis.

Participants were instructed to refrain from eating and drinking (except water) for 5 h before the blood collection. Venous blood samples were collected into ethylenediaminetetraacetic acid (EDTA) tubes. Samples were centrifuged for 10 min at 3000 × g at room temperature. After centrifugation, 1.10 ml of plasma was stored in a -80°C freezer in the presence of 1.10 μl of a 100 mM phenylmethanesulfonyl fluoride (PMSF) solution. PMSF is a serine protease inhibitor and prevents endocannabinoid breakdown by fatty acid amide hydrolase (FAAH). The PMSF solution was purchased from Merck. Due to logistic reasons, PMSF was not added to the plasma samples of 10 patients and 1 HC. This might not influence the results, as PMSF is added as a precaution in case blood cells would accidently leak FAAH during venipuncture. As blood cells can release anandamide, the concentration of anandamide measured in plasma depends on the time between blood collection and the separation of blood cells from the plasma (Vogeser *et al.*, 2006). We, therefore, aimed to store all samples in the -80°C freezer within 30 minutes after blood collection. In addition, as the concentrations of anandamide and 2-arachinoylglycerol (2-AG) fluctuate throughout the day (Hanlon, 2020, Hanlon *et al.*, 2016), we also strived to collect all blood samples in the late afternoon.

Plasma concentrations of anandamide and 2-AG were determined using previously published methods that are based on liquid chromatography coupled to tandem mass spectrometry (LC-MS/MS) (Balvers , Verhoeckx, & Witkamp, 2009, Balvers, Wortelboer, Witkamp, & Verhoeckx et al., 2013). In short, 1 mL of plasma was extracted with 4 mL of acetonitrile (ACN) containing 100 µM of PMSF and deuterated anandamide (AEA-d8) as an internal standard (Balvers *et al.*, 2009). The ACN extract was purified using solid phase extraction (SPE) on Bond Elut C8 SPE columns (Agilent, 200 mg). Columns were washed with 2 mL of 20% ACN in MQ water containing 0.10% trifluoroacetic acid (TFA). The analytes were eluted with 2 mL of 80% ACN in MQ water with 0.10% TFA, subsequently evaporated to dryness in a vacuum concentrator, and the extracts were stored at -80 °C until LC-MS/MS analysis (Balvers *et al.*, 2009). For 9 patients and 2 HC, there was not sufficient plasma (<1 mL) available for sample preparation. We, therefore, used an alternative method, protein precipitation (PP), that required only 0.10 mL of plasma (Balvers *et al.*, 2013). Both methods have equal performance, but the preference was given to the SPE-based method since this generally results in cleaner extracts which facilitate the analysis. 2-AG is sensitive to isomerization into 1-AG in an aqueous environment, and it is therefore known that LC-MS/MS analyses can reveal peaks for both 1-AG and 2-AG in plasma extracts, which can increase variability in 2-AG concentrations (Balvers *et al.*, 2013). Here, the combined 1-AG and 2-AG peaks were used to calculate the 2-AG concentrations.

The LC-MS/MS system consisted of an Acquity H-class ultra-pressure liquid chromatography system coupled to a Xevo TQ-S micro triple quadrupole mass spectrometer (Waters). Chromatographic separation of the extracts was achieved on an Acquity C8 BEH UPLC column (2.10 x 100 mm, 1.70 µm particle size; Waters) using a previously published gradient (Balvers *et al.*, 2013). Quantification was performed using six-point calibration curves using 1/x^2^ weighing. Data acquisition and processing were performed using MassLynx version 4.2 SCN 1001 software (Waters). Quality control (QC) samples were included in each analytical batch to monitor the analytical performance of the LC-MS/MS method. The inter-batch precision for unspiked QC plasma was < 10% for anandamide and 19% for the combined 1- and 2-AG peak. The intra-batch accuracy for spiked (with known amounts of analytes) QC samples was between 100 and 110% for anandamide and was 117% for 2-AG.

**Methods S3**. ^1^H-MRS acquisition and pre-processing.

Participants were asked to refrain from cannabis and alcohol during the period of 24 h before the MRI scan. All MRI images were obtained on a 3T scanner (Philips, Ingenia Elition X, Best, The Netherlands) with a 32-channel head coil at the Amsterdam UMC, the Netherlands. A structural whole-brain T1-weighted MRI scan was acquired for ^1^H-MRS voxel placement and brain tissue segmentation by use of a MPRAGE sequence ([repetition time [TR] = 7.00 ms; echo time [TE] = 3.20 ms; 180 slices; field of view [FOV] = 256 x 240 x 180 mm; voxel size = 1 x 1 x 1 mm; flip angle [FA] = 9°] or [TR = 9.00 ms; TE = 4.10 ms; 189 slices; FOV = 284 × 284 × 170 mm; voxel size = 0.90 × 0.90 × 0.90 mm; FA = 8°]). The concentration of Glx (glutamate + glutamine) was estimated using the Point Resolved Spectroscopy (PRESS) pulse sequence (TR = 2000 ms; TE = 35 ms; 128 averages; spectral width = 2000 Hz; number of points = 2048; voxel size = 30 x 20 x 20 mm, with MOIST water-suppression; Table S1), while the concentration of y-aminobutyric acid (GABA) was estimated using the Mescher-Garwood Point Resolved Spectroscopy (MEGA-PRESS) pulse sequence with an on- and off-resonance editing pulse applied at 1.90 ppm and 7.50 ppm, respectively (TR = 2000 ms; TE = 68 ms; 320 averages; spectral width = 2000 Hz; number of points = 2048; voxel size = 30 x 30 x 25 mm, with VAPOR water-suppression; Table S1) (Mescher, Merkle, Kirsch, Garwood, & Gruetter, 1998). For logistic reasons, two MEGA-PRESS sequences with similar scan parameters were used. Only the shimming method was different among the sequences. As it is difficult to differentiate glutamate and glutamine signals at 4T or lower magnetic field strengths (Liemburg *et al.*, 2016), we estimated Glx instead of glutamate concentrations. As in earlier studies, both ^1^H-MRS voxels were placed in the anterior cingulate cortex (ACC) parallel to the corpus callosum on the sagittal midline (Figure S2) (Iwata *et al.*, 2019).

PRESS spectra were analyzed by use of LCModel version 6.3-1P (Provencher, 1993, 2014) using the standard LC model basis set of 16 metabolites (i.e., aspartate, creatine, GABA, glucose, glutamate, glutamine, glycerophosphocholine, glycine, L-alanine, L-lactate, *myo*-inositol, N-acetylaspartate, N-acetylaspartylglutamate, phosphocholine, phosphocreatine, taurine). This basis set was acquired using the PRESS sequence at 3T with a TE of 35 ms. To limit the degree of freedom of the baseline spline and to control thereby the flexibility of the spline baseline, the DKNTMN parameter was set to 0.50, as recommended by Bhogal *et al.* (2017). Gannet version 3.1 (Edden *et al.*, 2014) in Matlab (The MathWorks, Inc., Natick, MA, 2016) was used to analyze the MEGA-PRESS spectra. As creatine levels might be altered in psychotic disorders (Merritt *et al.*, 2021), all metabolite estimates were referenced to water instead of creatine, similar to Egerton *et al.* (2022), Egerton *et al.* (2021). GABA concentrations reflect GABA plus macromolecules (GABA+) concentrations, as macromolecules resonate at the same frequency as GABA (3.00 ppm) (Rothman, Petroff, Behar, & Mattson, 1993).

The quality of all ^1^H-MRS spectra was visually checked. For Glx, spectra with a full-width at half-maximum (FWHM) ≥0.10 ppm (Li *et al.*, 2020) or a signal-to-noise ratio (SNR) that was 2 standard deviations below the mean SNR for the entire sample (i.e., SNR < 21.50) (Egerton *et al.*, 2021) were excluded from further analysis. In addition, if the Cramer-Rao lower variance bounds (CRLB) for Glx was ≥20% (Li *et al.*, 2020), the subject was removed from the corresponding analysis. For GABA+, exclusion criteria for the GABA+ peak were fit error (FE) ≥15% (Bojesen *et al.*, 2020), FWHM ≥0.10 ppm (Goldstein, Anderson, Pillai, Kydd, & Russell, 2015), or an SNR that was 2 standard deviations below the mean SNR for GABA+ of the entire sample (i.e., SNR < 10.40) (Egerton *et al.*, 2021). If one or more exclusion criteria were met, the metabolite data were excluded from further analyses. Spectral quality measures after quality control are reported in Table S2.

Finally, the T1-weighted MRI scan was used to extract the fractions of grey matter (GM), white matter (WM), and cerebrospinal fluid (CSF) in the PRESS and MEGA-PRESS voxels by use of Gannet version 3.1 (Edden *et al.*, 2014). All metabolite values were corrected for tissue composition of the voxel by use of Equation 1 (Egerton *et al.*, 2022, Merritt *et al.*, 2019). This equation takes into account the relative densities of water in the different compartments. In Equation 1, M_corr_ is the metabolite concentration in mM corrected for tissue fraction. M is the uncorrected metabolite concentration in mM. Lastly, WM, GM, and CSF correspond to the proportions of WM, GM, and CSF in the voxel, respectively. Average tissue fractions for the three compartments are reported in Table S2. ^1^H-MRS data of 12 patients and 16 HC are also reported in other as yet unpublished manuscripts.

M_corr_ = M*(WM + 1.21*GM + 1.55*CSF) / (WM + GM) (Equation 1)

**Methods S4**. PET acquisition and pre-processing.

Participants were asked to refrain from eating and drinking (except water) for 6 h, alcohol and cannabis for 24 h, and smoking for 3 h before the PET scan. One hour before the PET acquisition, all participants consumed 150 mg carbidopa (a peripheral aromatic acid decarboxylase inhibitor) and 400 mg entacapone (a peripheral catechol-O-methyltransferase inhibitor) to block peripheral metabolization of [^18^F]F-DOPA and decrease the formation of radiolabelled metabolites (Hoffman *et al.*, 1992, Sawle *et al.*, 1994). In addition, a low-dose computed tomography (CT) scan of the brain was obtained for attenuation correction purposes. Subsequently, approximately 185 MBq [^18^F]F-DOPA was administered as a single intravenous bolus injection. Immediately afterwards a 90-minute dynamic PET scan was made. PET data were obtained on a Siemens PET/CT system (Biograph mCT FlowTrue-V-128) (FOV = 256 x 256 mm; slice thickness = 2 mm; pixel spacing = 1.59 x 1.59 mm) and binned in 25 frames (5 x 1, 3 x 2, 3 x 3, and 14 x 5 minute[s]). A headrest and head straps were used to minimize neck and head movement during the PET scan.

PET data were reconstructed by use of an iterative reconstruction algorithm (5 iterations, 21 subsets) that corrects for the time of flight (TOF) and point spread function (PSF), followed by a 2 mm FWHM Gaussian smoothing filter. We visually inspected the PET images for movement with Vinci (v4.66; Max Planck Institute for Neurological Research, Cologne, Germany) (MPI for Metabolism Research, 2020, Vollmar *et al.*, 2003). Participants who moved >7.50 mm during the PET scan were excluded from further analyses, as attenuation correction might no longer be correct. PET and structural T1-weighted images were then co-registered, by use of Vinci, to a single PET frame acquired 7 minutes post-injection. This was based on mutual information and allowed us to correct for minor head movements (Čı́žek *et al.*, 2004). Afterwards, the T1-weighted images were segmented into GM, WM, and CSF by use of Statistical Parametric Mapping (SPM) 12 (Wellcome Centre for Human Neuroimaging, London, UK) and PVElab (v2.3; Neurobiology Research Unit, Copenhagen, Denmark) (Quarantelli *et al.*, 2004, Svarer *et al.*, 2005). As partial volume correction was part of the reconstruction method, this was not done by PVElab. The volumes of interest (i.e., striatum and cerebellum) were identified using the Hammers’ maximum probability atlas (Hammers *et al.*, 2003). Afterwards, Patlak graphical analysis (Patlak and Blasberg, 1985), which is implemented in PPET (Amsterdam UMC, Amsterdam, The Netherlands) (Boellaard, Yaqub, Lubberink, & Lammertsma, 2006), was used to calculate the influx constant *k*i^cer^ (min^-1^; henceforth described as *k*i^cer^) as a measure of striatal dopamine synthesis capacity, with the GM of the cerebellum as reference region. The cerebellum was used as a reference region (Hoshi *et al.*, 1993). Lastly, linear fitting was conducted on the PET images obtained between 25 and 90 minutes to acquire a whole-brain parametric image. From this parametric image, we extracted the *k*i^cer^ of the GM striatum. PET data of all subjects are reported in another paper (van Hooijdonk *et al.*, in press).*Supplementary tables*

**Table S1.** Description of MRS hardware, data acquisition, analysis, and quality assessment.

|  | **PRESS** | **MEGA-PRESS** |
| --- | --- | --- |
| **1. Hardware** |  |  |
| a. Field Strength | 3T | 3T |
| b. Manufacturer | Philips | Philips |
| c. Model | Ingenia Elition X | Ingenia Elition X |
| d. RF coil | 32-channel head coil | 32-channel head coil |
| e. Additional hardware | NA | NA |
| **2. Acquisition** |  |  |
| a. Pulse sequence | PRESS | MEGA-PRESS |
| b. VOI locations | ACC | ACC |
| c. Nominal VOI size | 30 x 20 x 20 mm | 30 x 30 x 25 mm |
| d. TR, TE | TR/TE = 2000/35 ms | TR/TE = 2000/68 ms |
| e. Total number of acquisitions/averages | 128 | 320 |
| f. Additional sequence parameters | 2000 Hz; 2048 data points | 2000 Hz; 2048 data points |
| g. Water suppression method | MOIST | VAPOR |
| h. Shimming method | 1^st^ order pencil beam | 1^st^/2^nd^ order pencil beam |
| i. Triggering or motion correction method | None | None |
| **3. Data analysis methods and outputs** |  |  |
| a. Analysis software | LC model 6.3-1P | Gannet 3.1 |
| b. Processing steps deviating from quoted product | 0.50 ppm DKNTMN |  |
| c. Output measure | Water scaled, partial volume corrected | Water scaled, partial volume corrected |
| d. Quantification references and assumptions | Default basis set | Default fitting |
| **4. Data quality** |  |  |
| a. Reported variables | SNR, FWHM | SNR, FWHM |
| b. Data exclusion criteria | SNR < 21.50^a^, FWHM **≥** 0.10 ppm, CRLB **≥** 20% | SNR < 10.40^b^, FWHM **≥** 0.10 ppm, FE **≥** 15% |
| c. Quality measures of post processing model fitting | CRLB | FE |
| d. Sample spectrum | Figure S1A | Figure S1B |

*Abbreviations:* ACC, anterior cingulate cortex; CRLB, Cramer-Rao lower variance bounds; DKNTMN, spline stiffness parameter; FE, fit error; FWHM, full width at half maximum; MEGA-PRESS, Mescher-Garwood Point Resolved Spectroscopy; NA, not applicable; PRESS, Point Resolved Spectroscopy; RF, radiofrequency; SNR, signal-to-noise ratio; TE, echo time; TR, repetition time; VOI, volume of interest; 3T, 3 Tesla.
^a^Spectra with a SNR that was 2 standard deviations below the mean SNR for the entire sample (i.e., SNR < 21.50) were excluded from further analysis.
^b^Spectra with a SNR that was 2 standard deviations below the mean SNR for the entire sample (i.e., SNR < 10.40) were excluded from further analysis.

**Table S2**. Spectral quality and tissue fraction measures^a^.

|  | **Patients with SSD (n=16)** | **Healthy controls (n=16)** | **p-value** |
| --- | --- | --- | --- |
| **PRESS** | **Mean (SD)** | **Mean (SD)** |  |
| CRLB Glx | 3.31 (0.60) | 3.38 (0.50) | 0.64 |
| FWHM | 0.03 (0.01) | 0.03 (0.01) | 0.72 |
| SNR | 27.31 (1.78) | 27.94 (2.35) | 0.25 |
| GM | 0.70 (0.03) | 0.70 (0.04) | 0.32 |
| WM | 0.15 (0.03) | 0.14 (0.03) | 0.27 |
| CSF | 0.168 (0.04) | 0.16 (0.03) | 0.87 |
|  | **Patients with SSD (n=17)** | **Healthy controls (n=16)** | **p-value** |
| **MEGA-PRESS** | **Mean (SD)** | **Mean (SD)** |  |
| FE GABA+ | 6.03 (1.72) | 5.21 (1.08) | 0.19 |
| FWHM GABA+ | 20.00 (1.48) | 19.40 (1.42) | 0.58 |
| SNR GABA+ | 17.24 (4.14) | 22.33 (3.76) | **0.001** |
| GM | 0.53 (0.03) | 0.54 (0.04) | 0.16 |
| WM | 0.36 (0.03) | 0.35 (0.04) | 0.31 |
| CSF | 0.12 (0.03) | 0.12 (0.02) | 0.85 |

Significant results are bold.

*Abbreviations*: CRLB, Cramer-Rao lower variance bounds; CSF, cerebrospinal fluid; GABA+, y-aminobutyric acid plus macromolecules; Glx, glutamate plus glutamine; GM, grey matter; FE, fit error; FWHM, full width at half maximum; MEGA-PRESS, Mescher-Garwood Point Resolved Spectroscopy; PRESS, Point Resolved Spectroscopy; SD, standard deviation; SNR, signal-to-noise ratio; SSD, schizophrenia spectrum disorder; WM, white matter. ^a^Group differences were assessed with the Mann-Whitney U test.

**Table S3**. Anandamide group differences.

| **Independent variables** | **Unstandardized beta coefficients** | **p-value** |
| --- | --- | --- |
| *Model 1*: Dependent variable: plasma concentration of anandamide, no outliers, R^2^ = 0.317, *F*(3, 20) = 2.96, p-value = 0.06 | | |
| Group^a^ | -0.08 [-0.16, -0.001] | **0.047** |
| Sex^b^ | -0.003 [-0.11, 0.10] | 0.95 |
| Cannabis lifetime use^c^ | -0.10 [-0.18, -0.02] | **0.02** |

Significant results are bold.

^a^Group was coded as null for healthy controls and one for patients. To examine the effects in the patient group, reversed coding for group was used.
^b^Sex was coded as null for males and one for females.

^c^Cannabis lifetime use was coded as null for less than six times and one for six or more times.

**Table S4**. Group differences after the exclusion of outliers.

|  | **Patients with SSD (n = 9)** | **Healthy controls (n = 14)** |  |
| --- | --- | --- | --- |
| **Imaging measures** | **Mean (SD)** | **Mean (SD)** | **p-value** |
| Striatal DSC in min-1 | 0.0152 (0.0017) | 0.0170 (0.0011) | **<0.01**^a^ |
|  | **Patients with SSD (n = 16)** | **Healthy controls (n = 14)** |  |
| **Blood measures** | **Mean (SD)** | **Mean (SD)** | **p-value** |
| 2-AG plasma concentration in ng/ml | 2.68 (0.93) | 2.49 (1.26) | 0.64^b^ |

Significant results are bold.

*Abbreviations*: DSC, dopamine synthesis capacity; standard deviation; SSD, schizophrenia spectrum disorder; 2-AG, 2-arachidonoylglycerol.

^a^Group differences were assessed with an independent t-test.
^b^Group differences were assessed with the Mann-Whitney U test.

**Table S5.** Multivariate linear regression analyses between imaging and blood measures without two patients that tested positive on cannabis^a^.

| **Independent variables** | **Unstandardized beta coefficients** | **p-value** |
| --- | --- | --- |
| *Model 1*: Dependent variable: Glx in the ACC, no outliers, subject with positive urine screening for cannabis excluded, R^2^ = 0.12, *F*(3, 26) = 1.15, p-value = 0.35 | | |
| Group^c^ | 1.39 [-1.80, 4.59] | 0.38 |
| Effect of anandamide plasma concentration in HC | -0.96 [-6.52, 4.61] | 0.73 |
| Effect of anandamide plasma concentration in patients | -5.86 [-12.57, 0.84] | 0.08 |
| Interaction term: Group x Anandamide plasma concentration | -4.90 [-13.61, 3.81] | 0.26 |
| *Model 2a*: Dependent variable: Glx in the ACC, outliers *not* excluded^b^, subject with positive urine screening for cannabis excluded, R^2^ = 0.09, *F*(3, 26) = 0.89, p-value = 0.46 | | |
| Group^c^ | 0.75 [-0.83, 2.33] | 0.34 |
| Effect of 2-AG plasma concentration in HC | -0.003 [-0.33, 0.32] | 0.99 |
| Effect of 2-AG plasma concentration in patients | -0.17 [-0.38, 0.05] | 0.12 |
| Interaction term: Group x Plasma concentration of 2-AG | -0.16 [-0.55, 0.23] | 0.40 |
| *Model 2b*: Dependent variable: Glx in the ACC, outliers excluded^b^, subject with positive urine screening for cannabis excluded, R^2^ = 0.26, *F*(3, 22) = 2.59, p-value = 0.08 | | |
| Group^c^ | 3.02 [0.65, 5.38] | **0.02** |
| Effect of 2-AG plasma concentration in HC | 0.25 [-0.22, 0.71] | 0.28 |
| Effect of 2-AG plasma concentration in patients | -0.85 [-1.54, -0.16] | **0.02** |
| Interaction term: Group x 2-AG plasma concentration | -1.09 [-1.93, -0.26] | **0.01** |
| *Model 3*: Dependent variable: GABA+ in the ACC, no outliers, subject with positive urine screening for cannabis excluded, R^2^ = 0.28, *F*(3, 27) = 3.47, p-value = **0.03** | | |
| Group^c^ | -0.60 [-1.82, 0.63] | 0.33 |
| Effect of anandamide plasma concentration in HC | -0.76 [-2.98, 1.45] | 0.49 |
| Effect of anandamide plasma concentration in patients | -0.85 [-3.15, 1.45] | 0.46 |
| Interaction term: Group x Anandamide plasma concentration | -0.09 [-3.28, 3.10] | 0.96 |
| *Model 4a*: Dependent variable: GABA+ in the ACC, outliers *not* excluded^b^, subject with positive urine screening for cannabis excluded, R^2^ = 0.36, *F*(3, 27) = 5.06, p-value = **0.007** | | |
| Group^c^ | -0.83 [-1.40, -0.26] | **0.006** |
| Effect of 2-AG plasma concentration in HC | -0.12 [-0.24, -0.002] | **0.046** |
| Effect of 2-AG plasma concentration in patients | -0.02 [-0.10, 0.06] | 0.60 |
| Interaction term: Group x 2-AG plasma concentration | 0.10 [-0.04, 0.25] | 0.15 |
| *Model 4b*: Dependent variable: GABA+ in the ACC, outliers excluded^b^, subject with positive urine screening for cannabis excluded, R^2^ = 0.41, *F*(3, 23) = 5.34, p-value = **0.006** | | |
| Group^c^ | -0.12 [-1.09, 0.86] | 0.81 |
| Effect of 2-AG plasma concentration in HC | -0.08 [-0.28, 0.12] | 0.43 |
| Effect of 2-AG plasma concentration in patients | -0.25 [-0.54, -0.03] | 0.08 |
| Interaction term: Group x 2-AG plasma concentration | -0.18 [-0.52, 0.17] | 0.31 |

Significant results are bold.

*Abbreviations*: ACC, anterior cingulate cortex; GABA+, y-aminobutyric acid plus macromolecules; Glx, glutamate plus glutamine; HC, healthy controls, 2-AG, 2-arachidonoylglycerol.

^a^Two patients had a positive drug screen for cannabis use on the day of the PET and/or ^1^H-MRS scan. We repeated the multivariate linear regression analyses without these subjects. As these subjects also substantially moved during the PET scan (and therefore excluded from the corresponding analyses), we only repeated the analyses with Glx and GABA+ concentrations in the ACC as dependent variable.
^b^For the PET data, one outlier was identified. For the 2-AG data, four outliers were identified.
^c^Group was coded as null for healthy controls and one for patients. To examine the effects in the patient group, reversed coding for group was used.

**Table S6.** Additional multivariate linear regression analyses with signal to noise ratio for GABA+ as covariate^a^.

| **Independent variables** | **Unstandardized beta coefficients [95%-CI]** | **p-value** |
| --- | --- | --- |
| *Model 1*: Dependent variable: GABA in the ACC, no outliers, R^2^ = 0.60, *F*(4, 26) = 9.53, p-value < **0.01** | | |
| Group^b^ | -0.11 [-1.07, 0.86] | 0.82 |
| Effect of anandamide plasma concentration in HC | -0.49 [-2.19, 1.20] | 0.55 |
| Effect of anandamide plasma concentration in patients | -0.89 [-2.65, 0.87] | 0.31 |
| Interaction term: Group x anandamide plasma concentration | -0.40 [-2.84, 2.05] | 0.74 |
| SNR GABA+ | 0.08 [0.04, 1.11] | **<0.01** |
| *Model 2*: Dependent variable: GABA in the ACC, outliers *not* excluded^c^, R^2^ = 0.61, *F*(4, 26) = 9.99, p-value < **0.01** | | |
| Group^b^ | -0.39 [-0.90, 0.12] | 0.13 |
| Effect of 2-AG plasma concentration in HC | -0.07 [-0.17, 0.03] | 0.15 |
| Effect of 2-AG plasma concentration in patients | -0.01 [-0.07, 0.06] | 0.84 |
| Interaction term: Group x 2-AG plasma concentration | 0.07 [-0.05, 0.18] | 0.25 |
| SNR GABA+ | 0.07 [0.03, 0.11] | **<0.01** |
| *Model 3*: Dependent variable: GABA in the ACC, outliers excluded^c^, R^2^ = 0.60, *F*(4, 22) = 8.38, p-value < **0.01** | | |
| Group^b^ | -0.05 [-0.87, 0.77] | 0.90 |
| Effect of 2-AG plasma concentration in HC | -0.01 [-0.18, 0.17] | 0.93 |
| Effect of 2-AG plasma concentration in patients | -0.09 [-0.35, 0.17] | 0.49 |
| Interaction term: Group x 2-AG plasma concentration | -0.08 [-0.38, 0.22] | 0.58 |
| SNR GABA+ | 0.06 [0.02, 0.11] | **0.003** |

Significant results are bold.

*Abbreviations*: ACC, anterior cingulate cortex; 2-AG, 2-arachidonoylglycerol; 95%-CI, 95% confidence interval.

^a^Two patients who had a positive drug screen for cannabis use on the day of the PET and/or ^1^H-MRS scan were excluded.

^b^Group was coded as null for healthy controls and one for patients. To examine the effects in the patient group, reversed coding for group was used.
^c^For the 2-AG data, four outliers were identified.

**Table S7. Additional multivariate linear regression analyses to examine the effect of covariates.**

| **Independent variables** | **Unstandardized beta coefficients [95%-CI]** | **p-value** |
| --- | --- | --- |
| *Model 1*: Dependent variable: Glx in the ACC, outliers excluded^a^, R^2^ = 0.32, *F*(4, 23) = 2.65, p-value = 0.06 | | |
| Group^b^ | 2.66 [0.03, 5.29] | **0.048** |
| Effect of 2-AG plasma concentration in HC | 0.12 [-0.42, 0.66] | 0.64 |
| Effect of 2-AG plasma concentration in patients | -0.92 [-1.58, -0.27] | **0.008** |
| Interaction term: Group x 2-AG plasma concentration | -1.04 [-1.89, -0.20] | **0.02** |
| Sex^c^ | -0.63 [-1.95, 0.70] | 0.34 |
| *Model 2*: Dependent variable: Glx in the ACC, outliers excluded^a^, R^2^ = 0.32, *F*(4, 23) = 2.72, p-value = 0.054 | | |
| Group^b^ | 3.23 [0.91, 5.55] | **0.009** |
| Effect of 2-AG plasma concentration in HC | 0.26 [-0.21, 0.73] | 0.26 |
| Effect of 2-AG plasma concentration in patients | -0.90 [-1.56, -0.25] | **0.009** |
| Interaction term: Group x 2-AG plasma concentration | -1.17 [-1.97, -0.37] | **0.006** |
| Age^d^ | 0.43 [-0.39, 1.24] | 0.29 |
| *Model 3*: Dependent variable: Glx in the ACC, outliers excluded^a^, R^2^ = 0.35, *F*(4, 23) = 3.07, p-value = **0.04** | | |
| Group^b^ | 3.75 [1.36, 6.14] | **0.004** |
| Effect of 2-AG plasma concentration in HC | 0.41 [-0.10, 0.93] | 0.11 |
| Effect of 2-AG plasma concentration in patients | -0.85 [-1.50, -0.20] | **0.01** |
| Interaction term: Group x 2-AG plasma concentration | -1.26 [-2.06, -0.47] | **0.003** |
| Cannabis lifetime use^e^ | -0.71 [-1.71, 0.29] | 0.16 |

Significant results are bold.

*Abbreviations*: ACC, anterior cingulate cortex; Glx, glutamate plus glutamine; 2-AG, 2-arachidonoylglycerol; 95%-CI, 95% confidence interval.

^a^For the 2-AG data, four outliers were identified.

^b^Group was coded as null for healthy controls and one for patients. To examine the effects in the patient group, reversed coding for group was used.
^c^Sex was coded as null for males and one for females.

^d^Age was coded as null for age 21 or younger and one for age 22 or older.

^e^Cannabis lifetime use was coded as null for less than six times and one for six or more times.

**Table S8.** Additional multivariate linear regression analyses to examine the effect of covariates without two patients that tested positive on cannabis^a^.

| **Independent variables** | **Unstandardized beta coefficients [95%-CI]** | **p-value** |
| --- | --- | --- |
| *Model 1*: Dependent variable: Glx in the ACC, outliers excluded^b^, subject with positive urine screening for cannabis excluded, R^2^ = 0.29, *F*(4, 21) = 2.18, p-value = 0.11 | | |
| Group^c^ | 2.44 [-0.23, 5.10] | 0.07 |
| Effect of 2-AG plasma concentration in HC | 0.12 [-0.42, 0.66] | 0.64 |
| Effect of 2-AG plasma concentration in patients | -0.85 [-1.54, -0.12] | **0.02** |
| Interaction term: Group x 2-AG plasma concentration | -0.97 [-1.84, -0.09] | **0.03** |
| Sex^d^ | -0.63 [-1.95, 0.70] | 0.34 |
| *Model 2*: Dependent variable: Glx in the ACC, outliers excluded^b^, subject with positive urine screening for cannabis excluded, R^2^ = 0.31, *F*(4, 21) = 2.31, p-value = 0.09 | | |
| Group^c^ | 3.05 [0.70, 5.39] | **0.01** |
| Effect of 2-AG plasma concentration in HC | 0.27 [-0.20, 0.73] | 0.25 |
| Effect of 2-AG plasma concentration in patients | -0.85 [-1.54, -0.17] | **0.02** |
| Interaction term: Group x 2-AG plasma concentration | -1.12 [-1.95, -0.29] | **0.01** |
| Age^e^ | 0.47 [-0.37, 1.31] | 0.26 |
| *Model 3*: Dependent variable: Glx in the ACC, outliers excluded^b^, subject with positive urine screening for cannabis excluded,R^2^ = 0.34, *F*(4, 21) = 2.72, p-value = 0.06 | | |
| Group^c^ | 3.63 [1.20, 6.05] | **0.005** |
| Effect of 2-AG plasma concentration in HC | 0.43 [-0.08, 0.94] | 0.10 |
| Effect of 2-AG plasma concentration in patients | -0.80 [-1.47, -0.13] | **0.02** |
| Interaction term: Group x 2-AG plasma concentration | -1.23 [-2.05, -0.40] | **0.005** |
| Cannabis lifetime use^f^ | -0.78 [-1.80, 0.24] | 0.13 |

Significant results are bold.

*Abbreviations*: ACC, anterior cingulate cortex; Glx, glutamate plus glutamine; 2-AG, 2-arachidonoylglycerol; 95%-CI, 95% confidence interval.

^a^Two patients had a positive drug screen for cannabis use on the day of the PET and/or ^1^H-MRS scan. We repeated the multivariate linear regression analyses without these subjects.

^b^For the 2-AG data, four outliers were identified.

^c^Group was coded as null for healthy controls and one for patients. To examine the effects in the patient group, reversed coding for group was used.
^d^Sex was coded as null for males and one for females.

^e^Age was coded as null for age 21 or younger and one for age 22 or older.

^f^Cannabis lifetime use was coded as null for less than six times and one for six or more times.

*Supplementary figures*


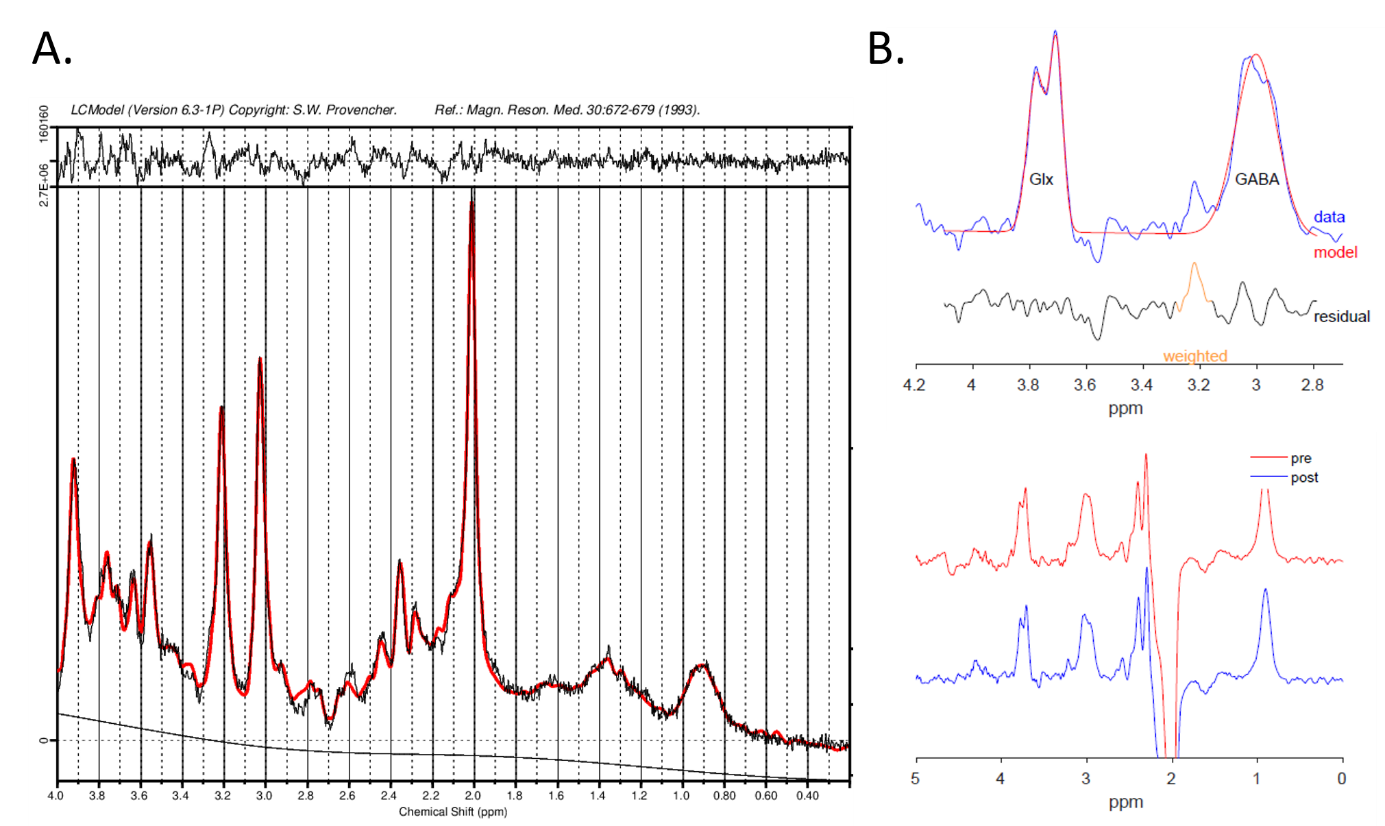


**Figure S1.** Example ^1^H-MRS spectra of one healthy control. (A) Representative PRESS spectrum fitted by LCModel (FWHM = 0.03, SNR = 30). (B) Representative MEGA-PRESS spectrum fitted by Gannet (FWHM = 18.32, FE = 4.90).
*Abbreviations*: FE, fit error; FWHM, full width at half maximum; GABA+, y-aminobutyric acid plus macromolecules; Glx, glutamate plus glutamine; MEGA-PRESS, Mescher-Garwood Point Resolved Spectroscopy; PRESS, Point Resolved Spectroscopy; SNR, signal-to-noise ratio; ^1^H-MRS, proton magnetic resonance spectroscopy.


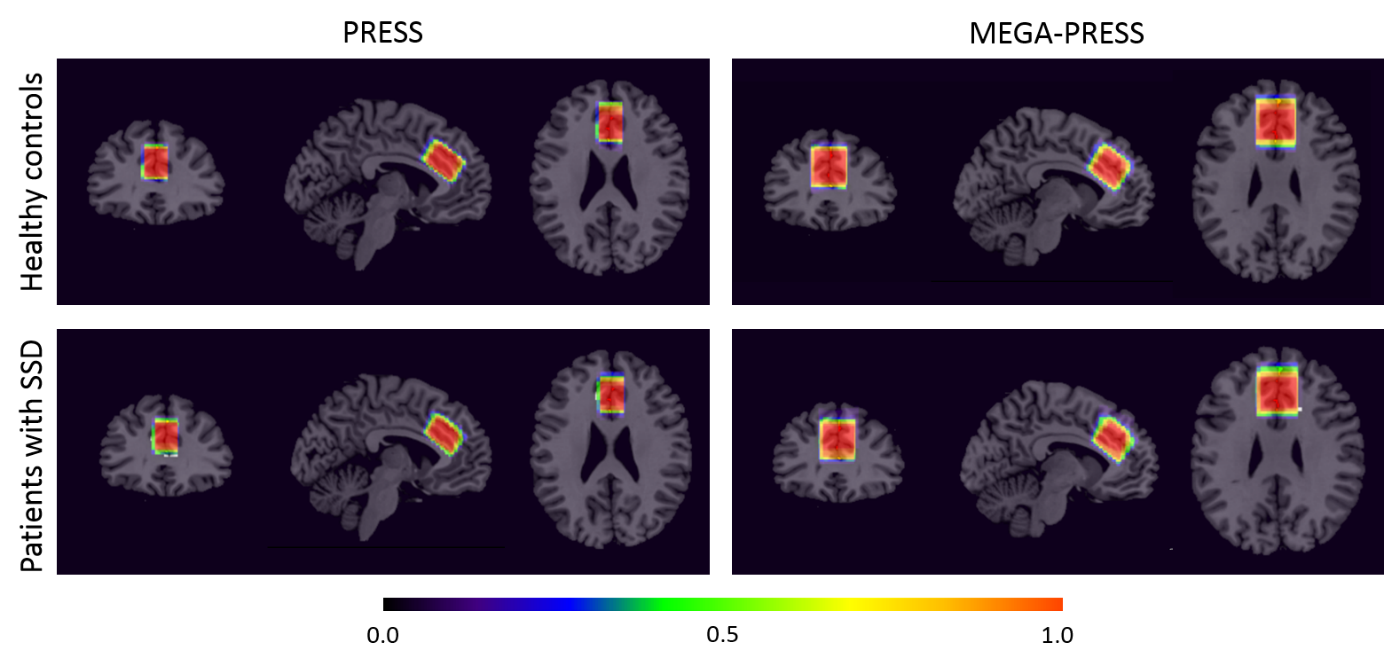
**Figure S2**. ^1^H-MRS voxel placement. Proton magnetic resonance spectroscopy (^1^H-MRS) voxel placement for PRESS (left view) and MEGA-PRESS scans (right view). Both voxels were placed in the anterior cingulate cortex (ACC) parallel to the corpus callosum on the sagittal midline. The colour bar demonstrates the proportion of voxel overlap between subjects within the healthy control (upper view) and patient groups (lower view), after normalization to MNI space. Red indicates greater overlap between subjects.
*Abbreviations*: MEGA-PRESS, Mescher-Garwood Point Resolved Spectroscopy; PRESS, Point Resolved Spectroscopy; SSD, schizophrenia spectrum disorder.

**
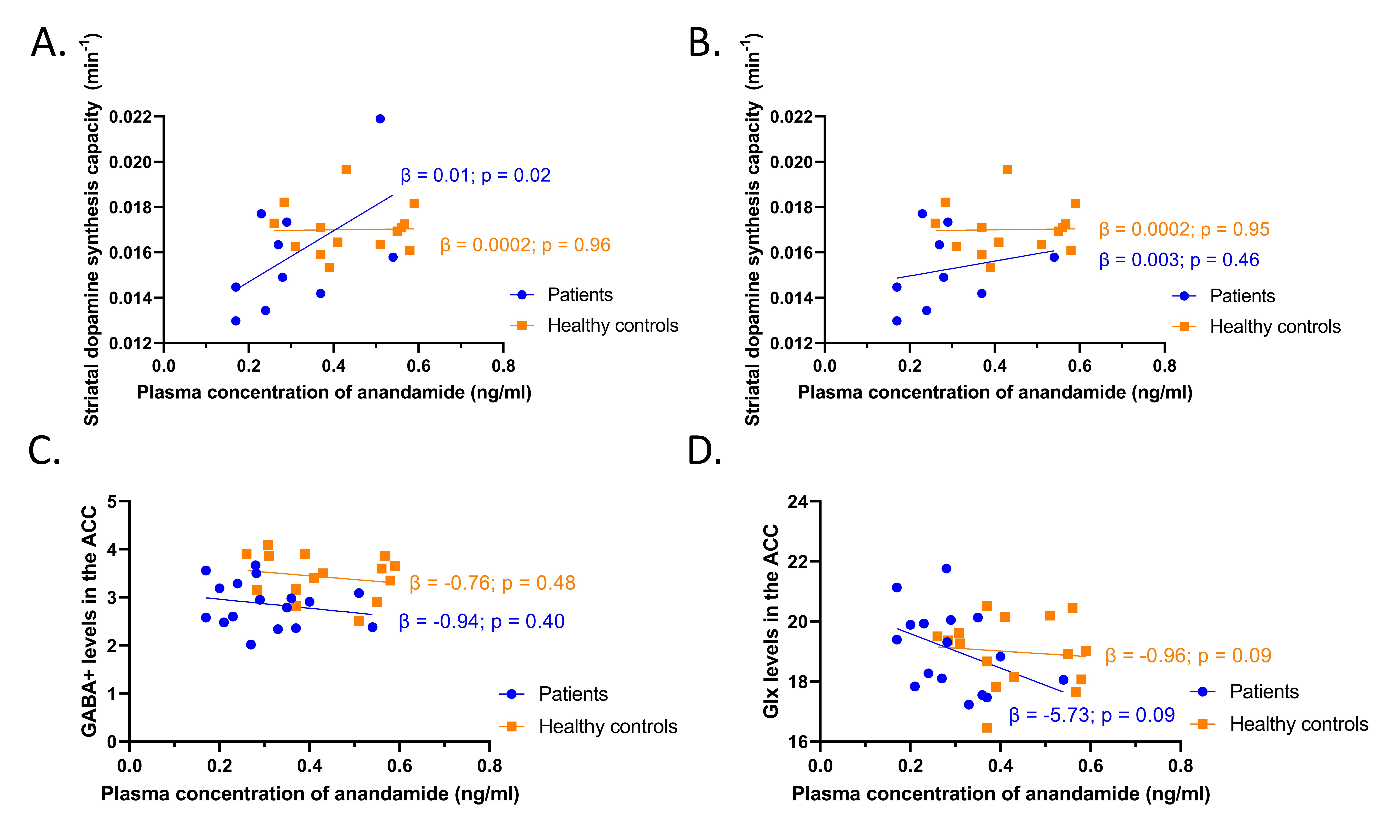
Figure S3.** Anandamide and imaging measures in patients with SSD and controls. Scatterplot displaying the correlation between plasma concentration of anandamide and striatal dopamine synthesis capacity with (A) and without (B) one outlier, GABA+ (C) and Glx levels in the ACC (D) in patients with SSD (blue circles) and healthy controls (orange squares).
*Abbreviations*: ACC, anterior cingulate cortex; GABA+, y-aminobutyric acid plus macromolecules; Glx, glutamate plus glutamine; SSD, schizophrenia spectrum disorders; β, unstandardized beta.

*
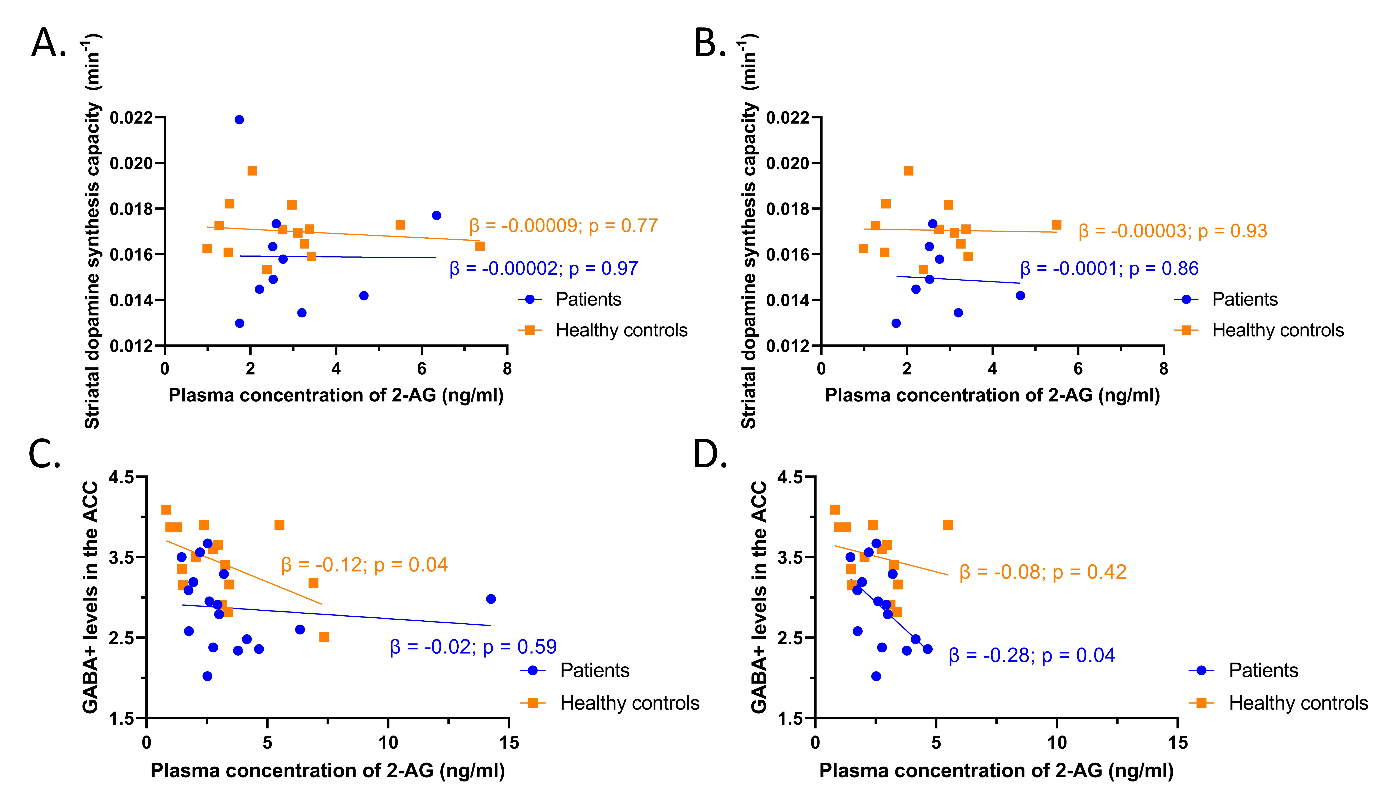
***Figure S4.** 2-AG and imaging measures in patients with SSD and controls. Scatterplot displaying the correlation between plasma concentration of 2-AG and striatal dopamine synthesis capacity with (A) and without (B) three outliers and GABA+ levels in the ACC with (C) and without four outliers in (D) in patients with SSD (blue circles) and healthy controls (orange squares).
*Abbreviations*: ACC, anterior cingulate cortex; GABA+, y-aminobutyric acid plus macromolecules; ; SSD, schizophrenia spectrum disorders; 2-AG, 2-arachidonoylglycerol; β, unstandardized beta.

*References*

Andreasen, N. C. (1987). *The Comprehensive Assessment of Symptoms and History (CASH)*. Department of Psychiatry, University of Iowa College of Medicine: Iowa City.

Balvers, M. G., Verhoeckx, K. C., & Witkamp, R. F. (2009). Development and validation of a quantitative method for the determination of 12 endocannabinoids and related compounds in human plasma using liquid chromatography-tandem mass spectrometry. *Journal of Chromatography* *B*, *877*(14–15), 1583–1590. doi:10.1016/j.jchromb.2009.04.010

Balvers, M. G., Wortelboer, H. M., Witkamp, R. F., & Verhoeckx, K. C. (2013). Liquid chromatography-tandem mass spectrometry analysis of free and esterified fatty acid N-acyl ethanolamines in plasma and blood cells. *Analytical Biochemistry, 434*(2), 275–283. doi:10.1016/j.ab.2012.11.008

Bhogal, A. A., Schür, R. R., Houtepen, L. C., van de Bank, B., Boer, V. O., Marsman, A., … Klomp, D. W. J. (2017). 1H-MRS processing parameters affect metabolite quantification: The urgent need for uniform and transparent standardization. *NMR in Biomedicine, 30*(11), e3804. doi:10.1002/nbm.3804

Boellaard, R., Yaqub, M., Lubberink, M., & Lammertsma, A. (2006). PPET: A software tool for kinetic and parametric analyses of dynamic PET studies. *NeuroImage, 31*(suppl 2), T62. doi:10.1016/j.neuroimage.2006.04.053

Bojesen, K. B., Ebdrup, B. H., Jessen, K., Sigvard, A., Tangmose, K., Edden, R. A., ... Glenthøj, B. Y. (2020). Treatment response after 6 and 26 weeks is related to baseline glutamate and GABA levels in antipsychotic-naïve patients with psychosis. *Psychological Medicine, 50*(13), 2182–2193. doi:10.1017/S0033291719002277

Čı́žek, J., Herholz, K., Vollmar, S., Schrader, R., Klein, J., & Heiss, W. D. (2004). Fast and robust registration of PET and MR images of human brain. *NeuroImage, 22*(1), 434–442. doi:10.1016/j.neuroimage.2004.01.016

Edden, R. A., Puts, N. A., Harris, A. D., Barker, P. B., & Evans, C. J. (2014). Gannet: A batch‐processing tool for the quantitative analysis of gamma‐aminobutyric acid-edited MR spectroscopy spectra. *Journal of Magnetic Resonance Imaging, 40(6)*, 1445–1452. doi:10.1002/jmri.24478

Egerton, A., Griffiths, K., Casetta, C., Deakin, B., Drake, R., Howes, O. D., ... MacCabe, J. H. (2022). Anterior cingulate glutamate metabolites as a predictor of antipsychotic response in first episode psychosis: Data from the STRATA collaboration. *Neuropsychopharmacology,* *48*(3), 567–575. doi:10.1038/s41386-022-01508-w

Egerton, A., Murphy, A., Donocik, J., Anton, A., Barker, G. J., Collier, T., ... Howes, O. D. (2021). Dopamine and glutamate in antipsychotic-responsive compared with antipsychotic-nonresponsive psychosis: A multicenter positron emission tomography and magnetic resonance spectroscopy study (STRATA). *Schizophrenia Bulletin, 47*(2), 505–516. doi:10.1093/schbul/sbaa128

Goldstein, M. E., Anderson, V. M., Pillai, A., Kydd, R. R. & Russell, B. R. (2015). Glutamatergic neurometabolites in clozapine-responsive and -resistant schizophrenia. *International Journal of Neuropsychopharmacology, 18*(6), pyu117. doi:10.1093/ijnp/pyu117

Hammers, A., Allom, R., Koepp, M. J., Free, S. L., Myers, R., Lemieux, L., … Duncan, J. S. (2003). Three‐dimensional maximum probability atlas of the human brain, with particular reference to the temporal lobe. *Human Brain Mapping, 19*(4), 224–247. doi:10.1002/hbm.10123

Hanlon, E. C. (2020). Impact of circadian rhythmicity and sleep restriction on circulating endocannabinoid (eCB) N-arachidonoylethanolamine (anandamide). *Psychoneuroendocrinology, 111*, 104471. doi:10.1016/j.psyneuen.2019.104471

Hanlon, E. C., Tasali, E., Leproult, R., Stuhr, K. L., Doncheck, E., De Wit, H., … Van Cauter, E. (2016). Sleep restriction enhances the daily rhythm of circulating levels of endocannabinoid 2-arachidonoylglycerol. *Sleep, 39*(3), 653–664. doi:10.5665/sleep.5546

Hoshi, H., Kuwabara, H., Léger, G., Cumming, P., Guttman, M. & Gjedde, A. (1993). 6-[18F] fluoro-L-dopa metabolism in living human brain: A comparison of six analytical methods. *Journal of Cerebral Blood Flow and Metabolism, 13*(1), 57–69. doi:10.1038/jcbfm.1993.8

Iwata, Y., Nakajima, S., Plitman, E., Caravaggio, F., Kim, J., Shah, P., ... Graff-Guerrero, A. (2019). Glutamatergic neurometabolite levels in patients with ultra-treatment-resistant schizophrenia: A cross-sectional 3T proton magnetic resonance spectroscopy study. *Biological Psychiatry, 85*(7), 596–605. doi:10.1016/j.biopsych.2018.09.009

Li, J., Ren, H., He, Y., Li, Z., Ma, X., Yuan, L., ... Tang, J. (2020). Anterior cingulate cortex glutamate levels are related to response to initial antipsychotic treatment in drug-naive first-episode schizophrenia patients. *Frontiers in Psychiatry, 11*, 553269. doi:10.3389/fpsyt.2020.553269

Liemburg, E., Sibeijn-Kuiper, A., Bais, L., Pijnenborg, G., Knegtering, H., van der Velde, J., … Aleman, A. (2016). Prefrontal NAA and Glx Levels in Different Stages of Psychotic Disorders: A 3T 1H-MRS Study. *Scientific Reports*, 6(1), 21873. doi:10.1038/srep21873

Merritt, K., McGuire, P. K., Egerton, A., 1H-MRS in Schizophrenia Investigators, Aleman, A., Block, W., ... Yamasue, H. (2021). Association of age, antipsychotic medication, and symptom severity in schizophrenia with proton magnetic resonance spectroscopy brain glutamate level: A mega-analysis of individual participant-level data. *JAMA Psychiatry, 78*(6), 667–681. doi:10.1001/jamapsychiatry.2021.0380

Merritt, K., Perez-Iglesias, R., Sendt, K.V., Goozee, R., Jauhar, S., Pepper, F., ... Egerton, A. (2019). Remission from antipsychotic treatment in first episode psychosis related to longitudinal changes in brain glutamate. *NPJ Schizophrenia, 5*(1), 12. doi:10.1038/s41537-019-0080-1

Mescher, M., Merkle, H., Kirsch, J., Garwood, M. & Gruetter (1998). Simultaneous in vivo spectral editing and water suppression. *NMR in Biomedicine, 11*(6), 266–272. doi:10.1002/(sici)1099-1492(199810)11:6<266::aid-nbm530>3.0.co;2-j

MPI for Metabolism Research (2020). VINCI. Retrieved from http://vinci.sf.mpg.de/

Patlak, C. S., & Blasberg, R. G. (1985). Graphical evaluation of blood-to-brain transfer constants from multiple-time uptake data. *Journal of Cerebral Blood Flow and Metabolism, 5*(4), 584–590. doi:10.1038/jcbfm.1983.1

Provencher, S. W. (1993). Estimation of metabolite concentrations from localized in vivo proton NMR spectra. *Magnetic Resonance in Medicine, 30*(6), 672–679. doi:10.1002/mrm.1910300604

Provencher, S. W. (2021, February 4). LCModel & LCMgui User’s Manual. Retrieved from http://www.lcmodel.ca/pub/LCModel/manual/manual.pdf

Quarantelli, M., Berkouk, K., Prinster, A., Landeau, B., Svarer, C., Balkay, L., … Salvatore, M. (2004). Integrated software for the analysis of brain PET/SPECT studies with partial-volume-effect correction. *Journal of Nuclear Medicine, 45*(2), 192–201. Retrieved from https://jnm.snmjournals.org/content/45/2/192.long

Rothman, D. L., Petroff, O., Behar, K. L., & Mattson, R. H. (1993). Localized 1H NMR measurements of gamma-aminobutyric acid in human brain in vivo. *Proceedings of the National Academy of Sciences, 90*(12), 5662–5666. doi:10.1073/pnas.90.12.5662

Sawle, G., Burn, D., Morrish, P., Lammertsma, A., Snow, B., Luthra, S., … Brooks, D. J. (1994). The effect of entacapone (OR‐611) on brain [18F]‐6‐L‐fluorodopa metabolism: Implications for levodopa therapy of Parkinson's disease. *Neurology, 44*(7), 1292–1297. doi:10.1212/wnl.44.7.1292

Sheehan, D. V., Lecrubier, Y., Sheehan, K. H., Amorim, P., Janavs, J., Weiller, E., … Dunbar, G. C. (1998). The Mini-International Neuropsychiatric Interview (MINI): The development and validation of a structured diagnostic psychiatric interview for DSM-IV and ICD-10. *Journal of Clinical Psychiatry, 59*(suppl 20), 22–33. Retrieved from https://www.psychiatrist.com/jcp/neurologic/neurology/mini-international-neuropsychiatric-interview-mini/

Svarer, C., Madsen, K., Hasselbalch, S. G., Pinborg, L. H., Haugbøl, S., Frøkjær, V. G., ... Knudsen, G. M. (2005). MR-based automatic delineation of volumes of interest in human brain PET images using probability maps. *NeuroImage, 24*(4), 969–979. doi:10.1016/j.neuroimage.2004.10.017

Ter Smitten, M., Smeets, R., & Van den Brink, W. (1998). *Composite International Diagnostic Interview (CIDI), Version 2.1*. World Health Organization: Amsterdam.

Van Hooijdonk, C. F. M., van der Pluijm, M., Smith, C., Yaqub, M., van Velden, F. H. P., Horga, G., … van de Giessen, E. (2023). Striatal dopamine synthesis capacity and neuromelanin in the substantia nigra: A multimodal imaging study in schizophrenia and healthy controls. *Neuroscience Applied, 2*, 101134*.* doi:10.1016/j.nsa.2023.101134

Vogeser, M., Hauer, D., Christina Azad, S., Huber, E., Storr, M. & Schelling, G. (2006). Release of anandamide from blood cells. *Clinical Chemistry and Laboratory Medicine, 44*(4), 488–491. doi:10.1515/CCLM.2006.065

Vollmar, S., Cizek, J., Sué, M., Klein, J., Jacobs, A. & Herholz, K. (2003). VINCI-volume imaging in neurological research, co-registration and ROIs included. *Forschung und Wissenschaftliches Rechnen, 2004*(114), 115–131. Retrieved from https://citeseerx.ist.psu.edu/document?repid=rep1&type=pdf&doi=d5e8890ea46f2df0c200615732693d28612c7ff3
